# Marker-trait association analyses revealed major novel QTLs for grain yield and related traits in durum wheat

**DOI:** 10.3389/fpls.2022.1009244

**Published:** 2023-01-26

**Authors:** Behailu Mulugeta, Kassahun Tesfaye, Rodomiro Ortiz, Eva Johansson, Teklehaimanot Hailesilassie, Cecilia Hammenhag, Faris Hailu, Mulatu Geleta

**Affiliations:** ^1^ Institute of Biotechnology, Addis Ababa University, Addis Ababa, Ethiopia; ^2^ Department of Plant Breeding, Swedish University of Agricultural Sciences, Alnarp, Sweden; ^3^ Sinana Agricultural Research Center, Oromia Agricultural Research Institute, Bale-Robe, Ethiopia; ^4^ Director General, Bio and Emerging Technology Institute (BETin), Addis Ababa, Ethiopia; ^5^ Department of Biology and Biotechnology, Wollo University, Dessie, Ethiopia

**Keywords:** candidate gene, durum wheat, GWAS, linkage disequilibrium, population structure, QTL

## Abstract

The growing global demand for wheat for food is rising due to the influence of population growth and climate change. The dissection of complex traits by employing a genome-wide association study (GWAS) allows the identification of DNA markers associated with complex traits to improve the productivity of crops. We used GWAS with 10,045 single nucleotide polymorphism (SNP) markers to search for genomic regions associated with grain yield and related traits based on diverse panels of Ethiopian durum wheat. In Ethiopia, multi-environment trials of the genotypes were carried out at five locations. The genotyping was conducted using the 25k Illumina Wheat SNP array to explore population structure, linkage disequilibrium (LD), and marker-trait associations (MTAs). For GWAS, the multi-locus Fixed and Random Model Circulating Probability Unification (FarmCPU) model was applied. Broad-sense heritability estimates were high, ranging from 0.63 (for grain yield) to 0.97 (for thousand-kernel weight). The population structure based on principal component analysis, and model-based cluster analysis revealed two genetically distinct clusters with limited admixtures. The LD among SNPs declined within the range of 2.02–10.04 Mbp with an average of 4.28 Mbp. The GWAS scan based on the mean performance of the genotypes across the environments identified 44 significant MTAs across the chromosomes. Twenty-six of these MTAs are novel, whereas the remaining 18 were previously reported and confirmed in this study. We also identified candidate genes for the novel loci potentially regulating the traits. Hence, this study highlights the significance of the Ethiopian durum wheat gene pool for improving durum wheat globally. Furthermore, a breeding strategy focusing on accumulating favorable alleles at these loci could improve durum wheat production in the East African highlands and elsewhere.

## 1 Introduction

Durum wheat (*Triticum turgidum*, L. var. *durum* Desf.) is a staple cereal crop produced to make pasta, bread, and other traditional food items (Sall et al., 2019; Ceglar et al., 2021). Durum wheat accounts for approximately 8% of global wheat production (Sall et al., 2019), and most of it (75%) is produced in the Mediterranean region (Xynias et al., 2020). The world’s largest producers are Turkey and Canada, while Ethiopia is the largest producer in Sub-Saharan Africa (SSA). Durum wheat was domesticated around 10,000 years ago in the Fertile Crescent (Özkan et al., 2002; Soriano et al., 2018; Ceglar et al., 2021) and has since then been a vital source of energy, minerals, and bioactive compounds in human nutrition (Johansson et al., 2020a). The durum wheat is an amphidiploid species containing an AABB genome, and its genome size is nearly 12 Gb (Maccaferri et al., 2019).

The current development of advanced DNA sequencing methods, functional genomic tools, and availability of different DNA chip technology has highly facilitated the genetic dissection of multi-genic traits of food crops (Collard and Mackill, 2008; Al-Khayri et al., 2016; Geleta and Ortiz, 2016). Association mapping (AM) has been widely used to dissect the genetic architecture behind traits like grain yield, host plant resistance to pathogens, drought and salinity tolerance, phenology, and quality traits (Maccaferri et al., 2010; Tuberosa, 2012; Canè et al., 2014; Turki et al., 2015; Giraldo et al., 2016; Mengistu et al., 2016; Kidane et al., 2019; Mérida-García et al., 2019; Mérida-García et al., 2020). Moreover, genome-wide association studies (GWAS) have been successfully used to map genetic loci and dissect the genomic regions underlying several vital traits in important food crops, such as barley (Bellucci et al., 2017; Borrego-Benjumea et al., 2021), and bread wheat (Li et al., 2019; Gao et al., 2021; Mekonnen et al., 2021).

In wheat, GWAS has been successfully applied to identify and dissect QTL associated with grain yield (Li et al., 2019; Gao et al., 2021), host plant resistance to pathogens (Alemu et al., 2021a; Alemui et al., 2021; Mekonnen et al., 2021), drought tolerance (Bhatta et al., 2018; Mathew et al., 2019), root architecture (Alemu et al., 2021b), phenology (Mekonnen et al., 2021), adaptation to salinity (Quamruzzaman et al., 2021), and end-use quality traits (Chen et al., 2019; Talini et al., 2020). However, in durum wheat, limited GWAS results have been reported across traits of interest, although some results are present for grain yield (Wang et al., 2019; Anuarbek et al., 2020), host plant resistance to pathogens (Liu et al., 2017b; Aoun et al., 2021), drought tolerance (Wang et al., 2019), root system architecture (Maccaferri et al., 2016; Alemu et al., 2021b), osmotic adjustment (Condorell et al., 2022), and phenology and quality traits (Fiedler et al., 2017). Furthermore, GWAS results reported on Ethiopian durum wheat cultivars and landraces are insufficient. Increased genomic research is needed to improve durum wheat production in Ethiopia by utilizing genomic-assisted breeding approaches.

Wheat landraces can be seen as an essential germplasm resource, with the potential to be utilized as a reservoir of crop diversity that harbors significant novel loci associated with agronomic, phenological, and end-use quality traits (Johansson et al., 2021). Landraces and their wild relatives have served as sources of valuable genes to improve modern cultivars for adaptation to diverse environments, grain yield, end-use quality, host plant resistance to the pathogen, and abiotic stress tolerance (Maccaferri et al., 2019; Johansson et al., 2020b; Sansaloni et al., 2020). Several reports revealed that Ethiopian durum wheat has high genetic diversity to be explored in the search for essential novel and valuable genes for improvements of traits such as grain yield, nutritional quality, host plant resistance to pathogens, and drought tolerance (Mengistu et al., 2016; Kabbaj et al., 2017; Kidane et al., 2019; Alemu et al., 2020a). Hence, understanding the genetic basis of these important traits using recent genomic-based research will facilitate the use of Ethiopian germplasm in an improvement program to maintain a food-secure future in the region.

This study aimed to use GWAS to define genomic regions in Ethiopian durum wheat associated with grain yield and related traits. Furthermore, population structure and linkage disequilibrium were evaluated for precise identification of the genetic basis of valuable genomic regions associated with grain yield and important agronomic traits.

## 2 Materials and methods

### 2.1 Germplasm

The present study used 420 Ethiopian durum wheat landraces and cultivars. To accommodate the extensive diversity of the Ethiopian durum wheat gene pool, 385 landraces were selected from different geographical regions of Ethiopia, while 35 were crossbred cultivars. **Supplementary Table 1** provides information on these landraces and cultivars. For simplicity, the landraces and cultivars will be designated as genotypes hereafter.

### 2.2 Description of test environment

The performance of the genotypes was evaluated across five locations in Ethiopia, namely, Akaki (AK; 09˚53’ 48” N/39˚49’ 16” E), Chefe Donsa (CD; 08˚58’ 57” N/39˚09’ 13” E), Holeta (HO; 09°01’ 15” N/38°28´ 26” E), Kulumsa (KU; 08°01’ 11” N/39°09’ 37” E) and Sinana (SN; 07°06´ 58” N/40°13´ 38” E) during the 2019 ˗ 2020 main crop-growing season. The testing locations represent the country’s major and most suitable durum wheat growing environments. The soil texture of each site is characterized as heavy clay for Akaki and Chefe Donsa and clay for Holeta, Kulumsa, and Sinana. The test sites are classified into ME (Mega environment)2:SW(Spring Wheat) high rainfall areas that receive more than 500 mm of rainfall during the crop growing cycle as defined by CIMMYT’s Wheat Breeding Program (Rajaram et al., 1994). Among the five test sites, Sinana, Kulumsa, and Holeta have been used by CIMMYT’s wheat breeding program targeting high potential environments in the highlands of East Africa. The Agro-ecology at the Akaki and Chefe Donsa sites are also similar to those at the other three test sites and are considered high-potential sites. During the crop-growing season, the mean monthly maximum and minimum temperature of the Sinana site ranged from 20.8°C to 23.9°C and 8.4°C to 9.2°C, respectively, with total rainfall of 810 mm (**Supplementary Table 2**). The Holeta site received a total rainfall of 852 mm, with the mean annual minimum and maximum temperature of 10°C and 24°C, respectively (**Supplementary Table 2**). The Chefe Donsa site received mean monthly minimum and maximum temperatures ranging from 9.4–11.8°C and 20.3–24.2°C, with a total rainfall of 870.5 mm. Whereas, the Akaki site received mean monthly minimum and maximum temperatures ranging from 9.89–13.82°C and 24.85–26.91°C, respectively, with a total rainfall amount of 711.5 mm. Kulumsa site received a total rainfall of 700 mm, with a mean monthly minimum temperature of 11°C and a mean monthly maximum temperature of 23°C.

### 2.3 Field experimental design

The experiment was laid out using an alpha lattice design with two replications containing 21 incomplete blocks with a block size of 20, according to Patterson and Williams (1976). The landraces and cultivars were randomly assigned and planted on a plot size of 1 m^2^ with 2.5 m x 0.4 m (two rows with 20 cm spacing). The space between the plots was 20 cm. A seed rate of 150 kg ha^−1^ and fertilizer rate of 50 kg N ha^-1^ and 100 kg of P_2_O_5_ ha^-1^ was applied to each plot. In order to maintain genotype uniformity (since the genotypes were mostly landraces with possible seed admixture), the genotypes were grown on different plots for two consecutive crop growing seasons (2017-2018) at Sinana agricultural research center, and individual plants that appeared to differ in any of the clearly visible phenotypic traits were removed.

### 2.4 Evaluation of phenotypic traits

In this study, phenotyping was conducted by applying the previously described methodology for evaluating wheat genetic resources (IBPGR, 1985). The traits measured were phenology (days to heading, days to physiological maturity, and grain filling period), plant architecture (plant height, spike length, and number of effective tillers per plant), grain yield, and grain yield-related traits (number of spikelets per spike, and thousand kernel weight).

### 2.5 Statistical analysis of the phenotypic data

Before further analysis, data were evaluated by the Shapiro–Wilk test to assess if they fit into the normal distribution. Furthermore, based on the results from the normality test, the homogeneity test was performed for the scored data in the experiment as described in Levene (1960). The R statistical software (R Development Core team, 2021) was used for computing descriptive statistics (mean, range, standard deviation), coefficient of variation, analysis of variance (ANOVA), correlation among traits, and broad-sense heritability. The linear mixed model (LMM) fitted by the Restricted/Estimated Maximum Likelihood method [REML, Corbeil and Searle (1976)] in R package “lme4” (Bates et al., 2015) was used to estimate the variance components of scored traits. To perform ANOVA for each test environment, the genotypes and blocks were considered fixed and random effects, respectively. The response of the i^th^ genotype in the j^th^ incomplete block with the l^th^ replication of each environment for a particular trait was described as:


(1) $${\text{Y}}_{\text{ijl}}=\text{µ}+{\text{τ}}_{\text{i}}+{\text{β}}_{\text{j}}+{\text{γ}}_{\text{l}\left(\text{j}\right)}+{\text{ξ}}_{\text{ijl}}$$

where Y_ijl_ is the phenotypic response of the i^th^ genotype in l^th^ incomplete block within j^th^ replication, µ is the overall mean, τ_i_ is the fixed effect of genotype i, β_j_ is the random effect of the j^th^ replicate, γ_l_ is the random effect of the j^th^ incomplete block nested in the l^th^ replication, and ξ_ijl_ is the residual error.

The combined ANOVA across environments inference was computed for all the response variables as follows:


$$Y_{ijlk}=\;µ\;+\tau_{i}+\beta_{l}+\gamma_{j}+E_{k}+\beta \gamma_{lj}+\gamma E_{jk}+\tau E_{ik}+\xi_{ijlk}\;\;\;\;\;\;\;\left(2\right)$$

where Y_ijlk_ is the observed phenotypic trait for i^th^ genotype in l^th^ incomplete block within j^th^ replication at the k^th^ environment, µ is the overall mean, τ_i_ is the fixed effect of genotype i, β_l_ is the random effect of j^th^ replication, γ_j_ is the random effect of the l^th^ incomplete block within j^th^ replication, E_k_ is the random effect of environment k, βγ_lj_ is random effect of incomplete block l nested in replication j, γE_jk_ is random effect of replication j in test environment k, τE_ik_ is random effect of interaction between genotype i and environment k, and ξ_ijlk_ is a random residual effect. For the sake of simplicity, we assumed that all the underlying random effects residuals are normally distributed with zero mean and are independent homoscedastic.

The best linear unbiased estimates (BLUEs) of measured traits for each genotype from each environment were obtained using META R software (Alvarado et al., 2020). The estimated means of BLUEs was used to compute the Pearson correlation coefficient (r) by the “cor. test” function in the R (R Development Core team, 2021) and GWAS analysis. The estimates of broad-sense heritability (H2) were computed from pooled ANOVA across environments (Gonçalves-Vidigal et al., 2008) as:


$$H^{2}=\frac{\sigma {\;}_{g}^{2}}{\left[\sigma {\;}_{g}^{2}+(\sigma {\;}_{gl}^{2}/l)+\left(\sigma {\;}_{e}^{2}/lr\right)\right]}\;\;\;\;\;\;\;\;\;\;\;\;\;\left(3\right)$$

where ${\text{σ }}_{\text{g}}^{2}$ is genotypic variance, $\sigma {\;}_{gl}^{2}$ is genotype by environment interaction variance, $\sigma {\;}_{e}^{2}$ is environmental variance, **l** is the number of environments, and **r** is the number of replications.

### 2.6 DNA extraction, genotyping, and filtering of SNP markers

A single spike representing each genotype was collected during field phenotyping for genotyping. Five healthy seeds from each spike were taken to represent each genotype and were planted in 3 L pots in a greenhouse at the Swedish University of Agricultural Science (SLU), Alnarp, Sweden. A total of ten 6 mm leaf discs sampled from five two-week-old seedlings of each genotype were collected in each well of a 96-deep well plate and freeze-dried using the CoolSafe ScanVAC Freeze Dryer according to the instructions provided by Trait Genetics. The freeze-dried samples in 96-well deep well plates were sent to TraitGenetics (GmbH, Gatersleben, Germany) for DNA extraction and subsequent genotyping. A standard cetyltrimethylammonium bromide (CTAB) protocol was used to extract DNA from the leaf samples in TraitGenetics’ lab. The 420 genotypes were genotyped using an Illumina Infinium 25k wheat single nucleotide polymorphism (SNP) array following the manufacturer´s protocol. The details of the SNP array can be found at https://www.traitgenetics.com/index.php/service-products. Based on a specific durum wheat cluster file developed by TraitGenetics that differentiates durum wheat from bread wheat, markers accurately scored for the A and B genomes were recorded.

Several criteria were used to filter the genotypic data obtained before further analysis. TASSEL 5.2.80 software (Bradbury et al., 2007) was used to remove SNP loci with missing data above 5% or with minor allele frequency (MAF) below 5% (including monomorphic loci). Further filtering of the remaining SNP loci was conducted based on the level of observed heterozygosity (Ho), and loci with Ho greater than 0.01 were excluded. These filtering steps resulted in 10,045 SNPs that were used for data analyses. The evaluation of these SNP loci showed that each of the 420 samples had less than 1% missing data, and hence no genotype was excluded from the data analyses.

### 2.7 Population structure and linkage disequilibrium (LD) analysis

The number of subgroups among the 420 genotypes was inferred by principal component analysis (PCA) and model-based clustering methods, which were computed by Genome Association and Integrated Prediction Tool (GAPIT) 3.0 (Wang and Zhang, 2021) and STRUCTURE 2.3.4. software (Pritchard et al., 2000; Falush et al., 2007), respectively. A Bayesian approach (MCMC: Markov Chain Monte Carlo) that assumes an ancestry model of ADMIXTURE and correlated allele frequencies among the subgroups was used for model-based cluster analysis. The length of the burn-in period was adjusted to 50,000, followed by 100,000 MCMC iterations for subgroups (K) ranging from one to ten. Ten independent runs were carried out for each K. The STRUCTURE results were visualized using STRUCTURE Harvester (Earl and vonHoldt, 2012). The number of best K was inferred using the delta K method described in Evanno et al. (2005). The optimum K value bar plot was drawn based on CLUMPAK online software (Kopelman et al., 2015).

Information on the pattern of linkage disequilibrium (LD) within a genetic material of interest is necessary to determine the marker density required for a genome-wide scan (Siol et al., 2017). Accordingly, LD was computed using TASSEL version 5.2.8 (Bradbury et al., 2007). The pairwise LD (squared allele frequency, r^2^) for pairs of SNP markers was computed according to Weir (1997). The intersection of the fitted curve with the cut-off threshold was considered the mean r^2^ value for each chromosome (Breseghello and Sorrells, 2006b; Liu et al., 2017c). The mean r^2^ value of each chromosome was computed and plotted against the chromosome’s physical distance. The physical distance at which the r^2^ value dropped to half its average maximum value was considered the LD decay rate (Huang et al., 2010). The r^2^ = 0.3 (*p<0.01*) was considered as a cut-off point to represent a limit of QTL between pairs of markers as indicated in previous studies for Ethiopian durum wheat panels (Liu et al., 2017c; Alemu et al., 2021b).

### 2.8 Identification of marker trait association

GWAS was conducted using best linear unbiased estimates (BLUEs) for nine phenotypic traits and 10,045 SNP markers to identify marker-trait association (MTA). The BLUEs for grain yield, spike length, and grain-filling period were calculated by considering days to heading (DTH) as a covariate in order to control the effect of heading time, as suggested in previous studies (Sabadin et al., 2012; Tuberosa, 2012). The analysis was performed by employing a multi-locus-based method, fixed and random model Circulating Probability Unification [FarmCPU, Liu et al. (2016)] model selection algorithm implemented in GAPIT 3 R package (Lipka et al., 2012; Tang et al., 2016; Wang and Zhang, 2021). The FarmCPU model algorithm eliminates potential confounding factors by employing the fixed and random effect models iteratively. This was done to overcome the overfitting model influences of the stepwise regression and to control spurious MTA caused by population structure and family relatedness. GAPIT 3 was also used to visualize the Manhattan and Quantile-quantile (QQ)-plots. The QQ-plot fits the model to account for the population structure.

The stringent false-positive discovery rate [FDR, *p<* 0.01 (Benjamini and Hochberg, 1995)] and Bonferroni-corrected threshold of (–log10 (0.05/n) = 5.30 was used, where n is the total numbers of SNPs) to declare a significant MTA between a marker and phenotypic trait. All MTAs above the threshold levels were rated as significant. The percentage of phenotypic variance explained (PVE) by individual MTA (Garcia et al., 2019) and a marker-based VanRaden kinship (K) matrix (VanRaden, 2008) for the genotypes of interest was also generated in R/GAPIT 3. It was assumed that an identified QTL is stable in the genomic region when significant MTA has appeared in two or more test locations, and the additive effects were concordant.

### 2.9 Identification of putative novel MTAs and associated candidate genes

The novelty of significant MTAs and their potential associated genes were determined by comparative analyses with previously published reports using different *Triticum* databases such as GrainGene, T3/wheat, and Wheat URGI (Alaux et al., 2018). The lists of different genes and functions were downloaded from the NCBI database (https://ftp.ncbi.nlm.nih.gov/genomes/all/GCA/900/231/445/GCA_900231445.1_Svevo.v1/) to identify genes related to significant MTAs. The nucleotide position extending from 1-5 cM up and downstream from the SNP position was used for searching the potential candidate genes, as previously reported for wheat (Breseghello and Sorrells, 2006a). The genes associated with the significant MTAs were obtained from the durum wheat (*Triticum turgidum* (Svevo.v1) reference genome) (Maccaferri et al., 2019).

## 3 Results

### 3.1 Phenotypic mean performance of genotypes

**Figure 1:**
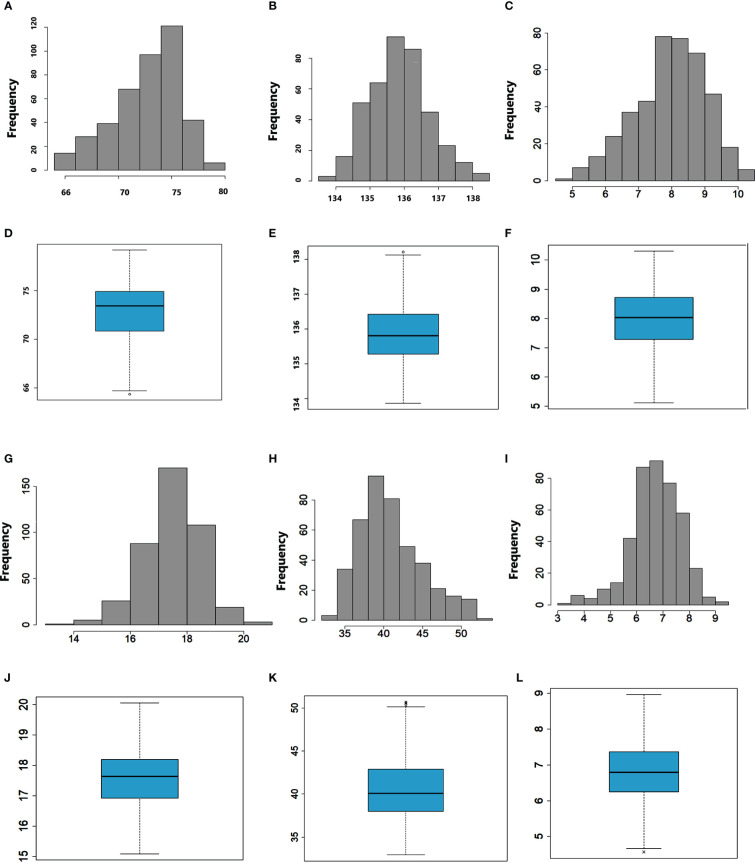
Frequency distribution and boxplots of DTH **(A, D)**, DTM **(B, E)**, SPL **(C, F)**, SPP **(G, J)**, TKW **(H, K)**, and GYD **(I, L)**.

Descriptive statistics, frequency distribution, and boxplots clearly showed a wide range of variation for all the traits evaluated (**Table 1** and **Figure 1**). The mean number of days for days to heading, days to physiological maturity, and grain filling period over the combined environments were 72.6, 136, and 63.4, respectively (**Table 1**). The mean grain yield was 6.7 t ha^-1^, while the mean thousand kernel weight was 40.9 g. On average, 17.5 spikelets per spike were recorded across the environments (**Table 1**). The highest mean grain yield (8.4 t ha^-1^) was observed at Chefe Donsa, followed by Sinana (8.2 t ha^-1^), whereas the lowest mean grain yield was recorded at Holeta (4.4 t ha^-1^) (**Table 1**). The highest mean performance of the genotypes for the thousand kernel weight (42.7 g) was attained at Chefe Donsa, whereas the lowest was found at Akaki (35.2 g). The pooled ANOVA over test environments indicated a significant (*p<0.01*) impact of genotype, environment, and genotype by environment interactions on all traits evaluated (**Supplementary Table 3**). Furthermore, significant effects of replications and blocks were noted for traits, most likely due to variation within the field.

**Table 1:** Descriptive statistics for days to heading (DTH, in days), days to physiological maturity (DTM, in days), grain filling period (GFP, in days), plant height (PHT, in cm), spike length (SPL, in cm), grain yield (GYD, in t ha^-1^), thousand kernel weight (TKW, in g), and the number of spikelets per spike (SPP, in counts) of 420 durum wheat genotypes grown in five test sites (ENV) in Ethiopia.

Traits	ENV	Mean	Median	Range	SE ^z^	Traits	ENV	Mean	Median	Range	SE
DTH	Akaki	77.3	78	59−86	0.19	SPL	Akaki	6.24	6	4−12	0.04
	Chefe Donsa	74.8	76	68−84	0.14		Chefe Donsa	7.69	8	5−14	0.05
	Holeta	74.9	76	64−84	0.14		Holeta	8.16	8	5−13	0.06
	Kulumsa	68.0	68	59−77	0.13		Kulumsa	8.1	8	5−12	0.05
	Sinana	64.1	68	59−76	0.13		Sinana	9.48	10	4−14	0.07
	Pooled ENV	72.6	73	59−86	0.08		Pooled ENV	7.94	8	4−14	0.03
DTM	Akaki	138.5	137	133−153	0.12	GYD	Akaki	4.9	4.9	1.2−9.9	0.05
	Chefe Donsa	137.3	138	131−150	0.09		Chefe Donsa	8.4	8.5	1.6−13	0.06
	Holeta	132.6	133	127−147	0.09		Holeta	4.36	4.3	2.7−11	0.03
	Kulumsa	133.6	134	127−145	0.09		Kulumsa	7.78	7.8	1.8−13.5	0.06
	Sinana	138.1	138	130−150	0.08		Sinana	8.2	8.1	2.9−14	0.07
	Pooled ENV	136.0	136	127−153	0.06		Pooled ENV	6.74	6.7	1.2−14	0.03
GFP	Akaki	61.3	60	48−81	0.17	TKW	Akaki	35.25	35	20−51	0.16
	Chefe Donsa	62.5	62	51−77	0.13		Chefe Donsa	42.69	42	27−60	0.18
	Holeta	57.7	57	48−75	0.15		Holeta	42.21	42	29−60	0.17
	Kulumsa	65.6	65	53−80	0.16		Kulumsa	42.25	42	26−66	0.20
	Sinana	70.0	70	58−85	0.14		Sinana	42.05	41.5	21−57	0.18
	Pooled ENV	63.4	63	48−85	0.09		Pooled ENV	40.89	41	20−66	0.09
PHT	Akaki	69.5	69	43−111	0.31	SPP	Akaki	15.84	16	9−23	0.07
	Chefe Donsa	100.0	100	70−133	0.33		Chefe Donsa	18.14	18	13−23	0.06
	Holeta	93.4	94	57−129	0.40		Holeta	14.58	15	10−23	0.05
	Kulumsa	101.2	102	58−140	0.37		Kulumsa	18.14	18	13−24	0.06
	Sinana	117.0	117	65−156	0.40		Sinana	20.88	21	14−26	0.06
	Pooled ENV	96.3	98	43−156	0.28		Pooled ENV	17.52	18	9−26	0.04

^z^SE, Standard error.

### 3.2 Variance components estimation, broad-sense heritability, and relationship between traits

The estimates of genotypic variance ( $\sigma_{g}^{2}$ ) and genotypic coefficient of variation (GCV) for the thousand kernel weight (TKW) and grain-filling period (GFP) were high. The lowest $\sigma_{g}^{2}$ and GCV were obtained for grain yield (GYD) and the number of effective tillers (NET; **Supplementary Table 4**). The highest values of variance due to genotype by environment interaction ( $\sigma_{gxe}^{2}$ ) and variance due to environments ( $\sigma_{gxe}^{2}$ ) were recorded for plant height (PHT). In contrast, the number of effective tillers per plant (NET) showed the lowest values of both variances. The phenotypic coefficient of variation (PCV) ranged from 24.1 for days to maturity (DTM) to 137.5 for TKW. Most of the phenotypic traits evaluated in the present study showed high heritability (**Supplementary Table 4**). The highest broad sense heritability values were recorded for TKW (H^2^ = 0.97) and GFP (H^2^ = 0.98), indicating that these traits are highly heritable.

**Figure 2:**
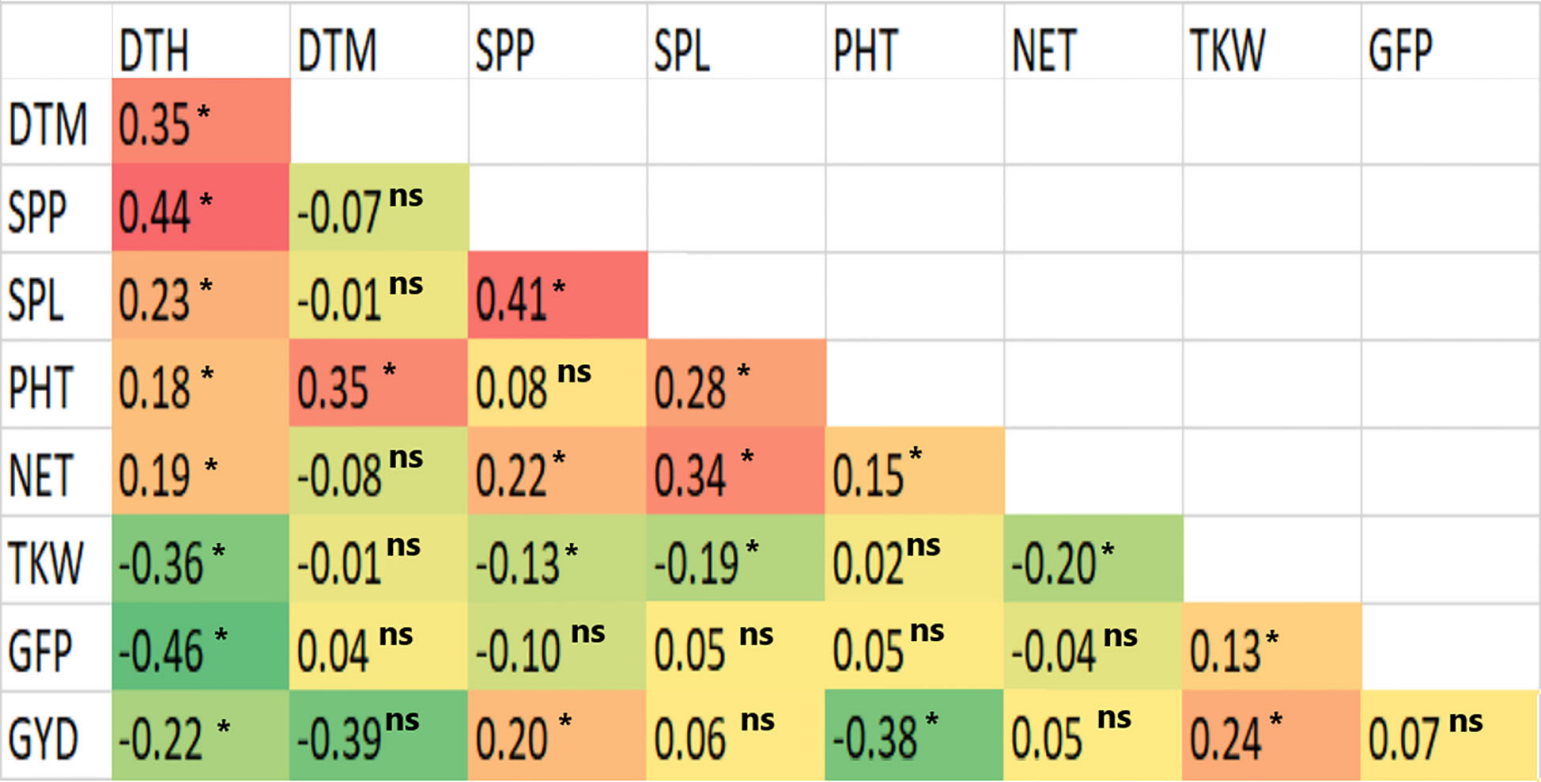
Computed correlation plots between pairs of phenotypic traits based on the best linear unbiased estimators of the nine traits measured in 420 durum wheat genotypes. N.B. *refers to significant at *p< 0.01*, and ns refers to non-significant at *p< 0.01*.

The Pearson correlation coefficients computed based on the BLUE mean values were positively significant (*p< 0.01*) for DTH with DTM, SPP, SPL, PHT and NET, for SPP with SPL, NET and GYD, and for SPL with PHT and NET (**Figure 2**). GYD was positively correlated with SPP (r = 0.20), and TKW (r = 0.24). Nevertheless, GYD had a negative correlation with DTH (r = -0.22) and PHT (r = -0.38) (**Figure 2**). DTH had a negative correlation with GFP and a positive correlation with DTM and SPP.

### 3.3 SNP markers distribution and density

In total, the 420 genotypes were genotyped with 24,145 SNP markers. Filtering of the genotypic data based on the number of missing values, observed heterozygosity, and minor allele frequency resulted in 10,045 high quality polymorphic SNPs used for data analyses. Of these 10,045 SNPs, 4,807 (48%) and 5,238 (52%) were distributed on the A and B genomes, respectively (**Table 2**). The number of these SNPs per chromosome with regard to the two genomes ranged from 415 on chromosome 4B to 917 on chromosome 5B (**Table 2**).

**Table 2:** The distribution of the 10,045 SNP markers across the entire durum wheat genome.

CHR^z^	NSPChr	GCR (bp)	SGRC (Mbp)	CHR	NSPChr	ROGC (bp)	SGRC (Mbp)
1A	757	1104472−585259074	584.2	1B	849	313555−681099620	680.8
2A	728	295475−774813964	774.5	2B	869	406084−789416853	789.4
3A	610	304055−746380464	746.1	3B	806	304239−746380464	746.1
4A	520	698412−736473645	735.8	4B	415	42526−674744571	674.7
5A	746	27537−667286510	667.3	5B	917	2555603−701346725	698.8
6A	643	591650−615260837	614.7	6B	753	2052283−698554772	696.5
7A	803	171878−727023089	726.9	7B	629	47368−721753586	721.7
A^a^	4807	na	4849.5	B^b^	5238	na	5008

^z^ CHR, chromosome; ^a^A, A genome; ^b^B, B genome; NSPChr, Number of SNPs per chromosome; GCR, Genome coverage range; SGRC, Size of genomic region(s) covered; na, Not applicable.

**Figure 3:**
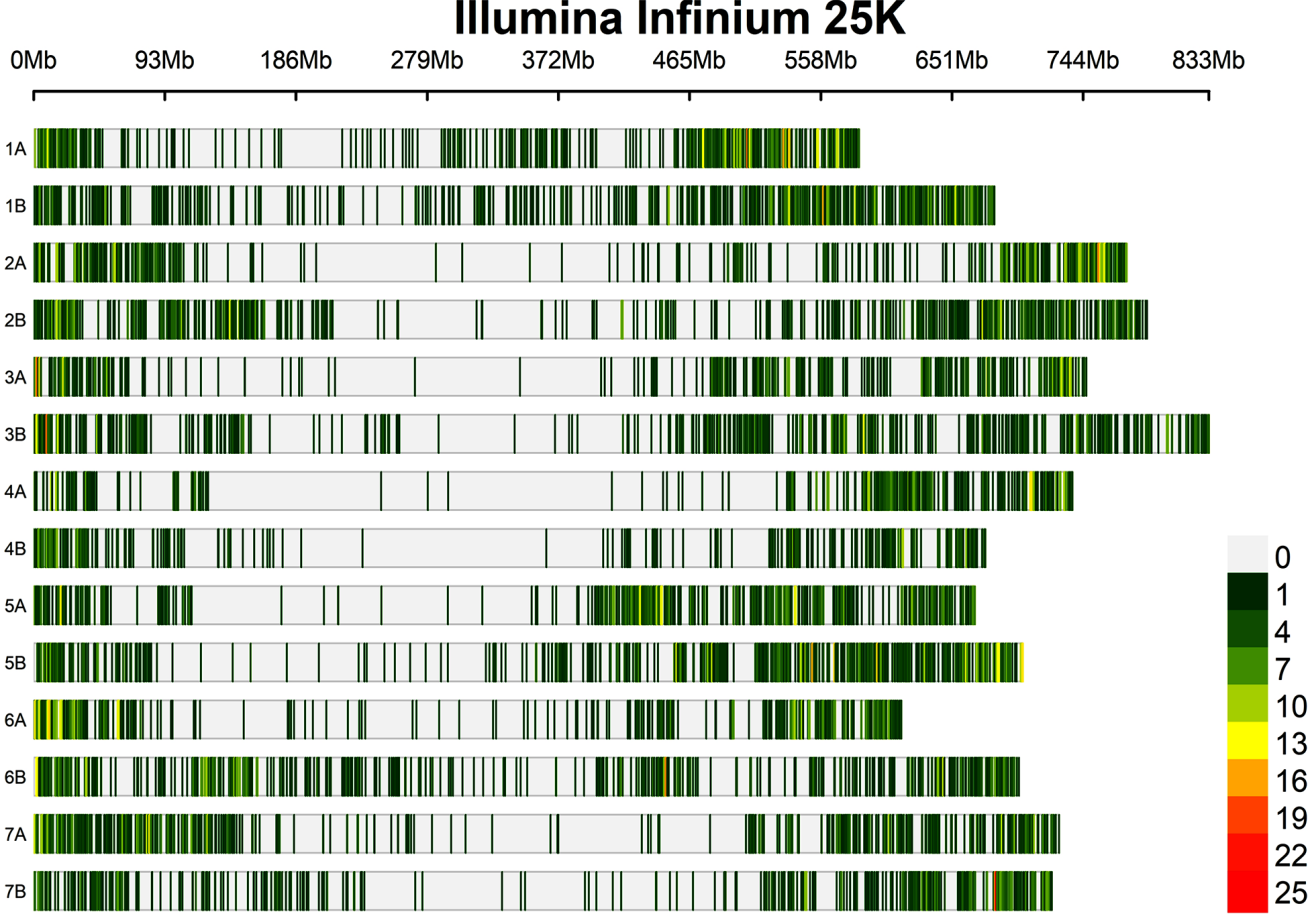
Distribution of the SNPs used in the present study on each durum wheat chromosome.

The marker density was 1.01, 0.96, and 0.98 Mbp per marker for the A, B, and whole genomes, respectively. The SNP markers used in this study covered a total size of 9.86 Gbps, with chromosomes 1A and 2B having the smallest (584.2 Mbp) and largest (789.4 Mbp) regions (**Table 2** and **Figure 3**).

### 3.4 Linkage disequilibrium

Among all possible pairs of SNPs on each chromosome, 490,775 pairs were found in LD (**Table 3**). Of these, 97,386 (19.8%) were found to be significant marker pairs with r^2^ ≥ 0.3 (*p*< 0.01; **Table 3**), which were therefore used to assess the MTAs. The significant marker pairs on each chromosome accounted for 12.1% (r^2^ = 0.12 for chromosome 7A) to 26.2% (r^2^ = 0.211 for chromosome 3B) of all marker pairs on the corresponding chromosomes (**Table 3**). The sudden LD decay among SNP pairs occurred within the range of 2.02–10.04 Mbp with an average of 4.26 Mbp (**Supplementary Table 5** **;** **Supplementary Figure 1**). The fastest decrease of LD at cut-off (r^2^ = 0.3) was observed on chromosome 7A. The r^2^ values of marker pairs progressively declined as the physical distance between them increased on each chromosome (**Supplementary Figure 1**).

**Table 3:** A summary of linkage disequilibrium analysis for SNP marker pairs and the distribution of significant SNP pairs across each chromosome of each genome.

Chromosome	Total number of SNP pairs	Significant SNP marker pairs at r^2^ ≥ 0.3 (*p<* 0.01)	Average r^2^	Average distance (Mbp ^z^)
1A	35,875	7,811 (21.8%)	0.20	20.4
1B	41,700	7,666 (18.4%)	0.16	21.1
2A	35,700	7,218 (20.2%)	0.17	28.3
2B	42,700	8,709 (20.4%)	0.17	23.8
3A	29,800	5,297 (17.8%)	0.16	32.9
3B	39,550	10,362 (26.2%)	0.21	27.4
4A	25,250	3,580 (14.2%)	0.14	38.4
4B	20,000	3,805 (19.0%)	0.17	44.9
5A	36,600	8,144 (22.3%)	0.19	23.7
5B	45,100	8,738 (19.4%)	0.17	20.1
6A	31,450	8,454 (26.9%)	0.22	25.6
6B	36,900	7,586 (20.6%)	0.18	24.6
7A	39,450	4,781 (12.1%)	0.12	23.9
7B	30,700	5,234 (17.0%)	0.15	30.8
A genome	234,125	45,284 (19.3%)	0.17	26.9
B genome	293,250	52,102 (17.8%)	0.17	25.8
Total	490,775	97,386 (19.8%)	0.17	26.8

^z^ Mbp, Mega base pair.

### 3.5 Principal component analysis, population structure, and kinship

**Figure 4:**
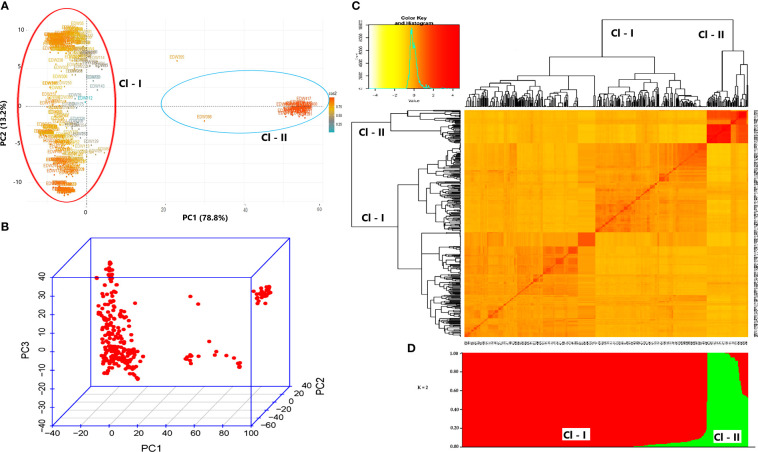
Principal component analysis **(A, B)**, kinship **(C)**, and population structure analysis **(D)**.

The PCA scatter plot explained 92% (PC1 = 78.8% and PC2 = 13.2%) of the entire variation in the data set and grouped the genotypes into two subpopulations (**Figure 4A**). Subpopulation 1 contained almost all modern cultivars, and subpopulation two included all landraces by showing clear grouping based on genetic background (**Figures 4A, B**).

A model-based Bayesian cluster analysis using STRUCTURE revealed that the optimal uppermost clear true ΔK value was obtained at best when K = 2, suggesting the 420 genotypes form two subpopulations (**Figure 4D**). Based on this clustering, cluster-1 comprised 348 landraces and one cultivar (85.5% of the genotype), and cluster-2 comprised 33 cultivars and 28 landraces (14.5% of the genotype). Analysis of admixture and purity using the STRUCTURE based on the Q value score (Q< 0.80 = admixture, and Q > 0.80 = pure genotypes) revealed that only 17 individuals (16 landraces and 1 cultivar) were classified as admixtures.

The STRUCTURE analysis revealed that 27 landraces were grouped with cultivars, and of these, three landraces (G242, G243, and G368) had a Q value of 1, which indicates 100% fitting the grouping with modern cultivars. Furthermore, one modern cultivar (G405), which was derived from related to landraces (according to their pedigree information), was grouped with landraces. The allelic divergence between the two subpopulations inferred by STRUCTURE was 0.27. On average, the expected heterozygosity of subpopulation-1 (Cl - I) and subpopulation-2 (Cl - II) was 0.22 and 0.32, respectively. Subpopulation-1 had a mean F_ST_ value of 0.62, while subpopulation-2 had a mean F_ST_ value of 0.35, indicating high differentiation among the individuals of each population. Although a slight difference was observed, model-based Bayesian clustering and distance-based PCA similarly grouped the individuals into two subpopulations. The kinship matrix heatmap revealed familial relationships between the genotypes, which can be regarded as intermediate on average (**Figure 4C**).

### 3.6 GWAS scan of phenotypic traits

Considering all test locations and combined data over locations, GWAS was able to identify 179 significant MTAs for the nine traits (**Supplementary Table 6**). Of these, 23 MTAs were detected for DTH, 32 MTAs for DTM, 15 MTAs for GFP, 8 MTAs for GYD, 5 MTAs for NET, 19 MTAs for PHT, 26 MTAs for spike length (SPL), 12 MTAs for SPP and 39 MTAs for TKW. Using BLUEs of combined data across the five environments revealed 44 significant MTAs for the nine traits evaluated in this study. Further results and discussions (below) focus on these significant MTAs identified using the combined data across the five environments. The Manhattan and quantile-quantile (Q-Q) plots for each trait and environment are presented in **Supplementary Figures 2A-E**, respectively.

#### 3.6.1 Marker trait association for phenological traits

**Figure 5:**
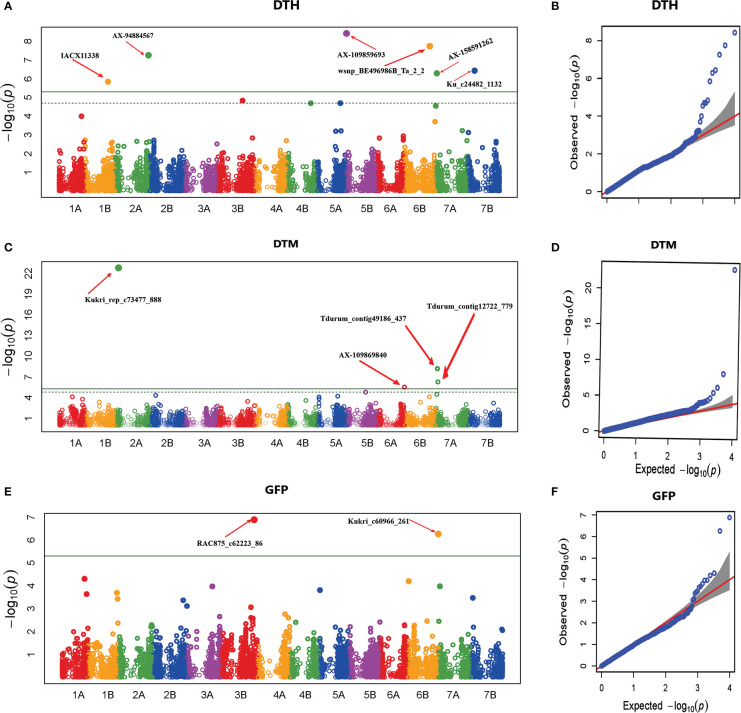
Manhattan and Q-Q plots of GWAS scan for phenological traits generated based on combined data from five locations. DTH **(A, B)**, DTM **(C, D)**, and GFP **(E, F)**. For the Manhattan plots, the y-axis represents -log10 (p) of the traits, while the x-axis represents the relative positions of the SNP markers on each chromosome. DTH, Days to heading; DTM, Days to maturity; GFP, Grain-filling period.

For phenological traits (DTH, DTM, and GFP), 12 significant MTAs were identified from the GWAS of combined data from the five locations (**Table 4**). The GWAS scan for DTH detected six significant MTAs on chromosomes 1B, 2A, 5B, 6B, 7A, and 7B (**Figure 5** and **Table 4**). The Q-Q plot showed that the data fitted the model well, and false positive MTAs were controlled. Among these MTAs, three were previously reported (Golabadi et al., 2011; Giraldo et al., 2016; Mangini et al., 2018), while three MTAs on the B genome (AX-109859693, wsnp_BE496986B_Ta_2_2, Ku_c24482_1132) were novel. The significant MTAs explained 1.1 to 22.2% of the total phenotypic variation in DTH. Among the significant MTAs for DTH, wsnp_BE496986B_Ta_2_2, AX-109859693, Ku_c24482_1132, and IACX11338 appeared significant in two or more test environments and hence can be regarded as stable MTAs.

**Table 4:** Summary of significant marker-trait associations for the nine traits revealed based on the combined data of the five locations on each durum wheat chromosome (CHR).

SNP (MTAs)	CHR	POS (bp)	P-value	MAF	Effect		PVE^Z^	Trait
AX-158591262	7A	30075367	5.13281E-07	0.31	-0.51		1.62	DTH
AX-94884567	2A	756029643	5.54677E-08	0.16	0.77		1.1	
IACX11338	1B	522669860	1.43456E-06	0.09	-2.04		22.18	
AX-109859693	5B	5172617	3.78017E-09	0.05	1.16		5.13	
wsnp_BE496986B_Ta_2_2	6B	568039035	1.80668E-08	0.17	-0.68		5.28	
Ku_c24482_1132	7B	155935903	3.69626E-07	0.29	-0.66		1.67	
AX-109869840	6A	603461435	2.75361E-06	0.05	-0.74		21.07	DTM
Kukri_rep_c73477_888	2A	70572673	1.55589E-23	0.09	-2.54		32.88	
Tdurum_contig49186_437	7A	32558067	5.98292E-09	0.49	0.29		1.88	
Tdurum_contig12722_779	7A	44540113	4.95657E-07	0.07	0.41		4.45	
RAC875_c62223_86	3B	763192473	1.31126E-07	0.32	-0.53		3.58	GFP
Kukri_c60966_261	6B	693337622	5.50108E-07	0.08	-1.41		30.67	
RFL_Contig3481_1669	1B	4043159	5.87000E-06	0.10	0.02		5.45	GYD
Excalibur_c51720_84	7A	709197555	6.71000E-06	0.05	0.02		15.75	
RAC875_c57656_170	7A	614197852	1.75039E-06	0.46	0.17		1.74	
IAAV3365	5A	548344620	1.87701E-10	0.06	-0.79		44.95	
GENE-0410_71	1B	523053033	1.78840E-07	0.44	0.22		5.61	NET
AX-94782013	7B	604310198	3.10157E-06	0.09	-0.25		2.79	
AX-158602974	1B	580658793	5.16606E-11	0.06	-4.33		2.68	PHT
BS00091519_51	5B	5174649	2.79112E-06	0.05	-2.03		9.64	
AX-158521163	1B	669849432	3.25148E-06	0.06	-2.10		1.2	
AX-95259256	1B	629504430	2.07679E-07	0.12	-1.52		4.51	
wsnp_BE443745A_Ta_2_1	5A	439542987	7.51027E-08	0.37	1.12		2.59	
AX-95154560	6B	120830636	5.51278E-08	0.05	2.73		23.76	
Tdurum_contig75127_589	7B	697951769	5.0922E-08	0.06	-3.88		8.34	
Tdurum_contig45715_1246	1B	314321199	6.74631E-07	0.48	-0.26		3.62	SPL
Kukri_c17062_618	2A	522595273	4.96382E-07	0.28	0.17		2.75	
Tdurum_contig76960_213	2A	492195805	3.17135E-07	0.13	-0.63		12.3	
Kukri_c3096_1411	2B	314134332	3.40111E-07	0.22	0.28		3.02	
AX-94615777	5A	529858969	2.51717E-07	0.45	0.15		1.01	
Tdurum_contig25602_212	2B	546442999	3.25386E-07	0.24	0.22		1.76	SPP
AX-158591111	7A	33518205	7.03418E-08	0.24	-0.24		1.85	
BS00110281_51	4A	724872914	1.51333E-06	0.06	0.41		4.5	
AX-89760660	1B	519060573	8.65163E-09	0.07	-0.49		16.82	
RAC875_c400_193	1B	1547605	8.21951E-07	0.10	-0.25		1.53	
AX-158597411	2B	99223728	2.38152E-06	0.49	0.24		2.27	
AX-94631122	3A	723577013	1.23025E-09	0.23	0.31		1.12	
AX-158606713	1B	546979073	1.26545E-06	0.26	-0.67	1.05		TKW
wsnp_Ex_rep_c66939_65371026	7A	6480158	1.99623E-06	0.18	-0.81	1.72		
BS00071597_51	3B	803879943	7.43197E-09	0.20	1.44	7.93		
AX-158541767	3B	61267921	2.83169E-07	0.07	-1.82	10.6		
RAC875_c41315_157	5A	431829169	3.78422E-06	0.10	-1.28	5.65		
AX-158564275	6A	528989018	3.23204E-10	0.09	-1.57	5.45		
AX-94640059	7A	686968079	4.86263E-06	0.44	0.65	1.23		

Chr, Chromosome; POS, Physical position of SNP; bp, Base pair; MAF, Minor allele frequency; PVE, Phenotypic variance explained; DTM, Days to heading; DTM, Days to maturity; GFP, Grain filling period; NET, Number of effect tillers per plant; SPL, Spike length; PHT, Plant height; SPP, Number of spikelets per spike; TKW, Thousand kernel weight; GYD, Grain yield.

The GWAS scan detected four significant MTAs across test environments for DTM. Of these MTAs, Kukri_rep_c73477_888 on chromosome 6A was previously reported (Mangini et al., 2018) and was detected in two environments. Three MTAs (Tdurum_contig49186_437 and Tdurum_contig12722_779 on chromosome 7A; and AX-109869840 on chromosome 6A) were likely to be potential new loci (**Table 4** and **Figure 5**). The proportion of phenotypic variance explained by these four significant SNPs ranged from 2 to 33%.

GWAS revealed two significant MTAs for GFP on chromosomes 3B and 7B (**Table 4** and **Figure 5**). The RAC875_c62223_86 MTA on 3B was previously reported (Giraldo et al., 2016). However, Kukri_c60966_261 on chromosome 7B was novel and detected repeatedly in two locations. RAC875_c62223_86 and Kukri_c60966_261 explained 4% and 31% of the variation in GFP obtained in the present study, respectively.

#### 3.6.2 Marker trait association for plant architecture

**Figure 6:**
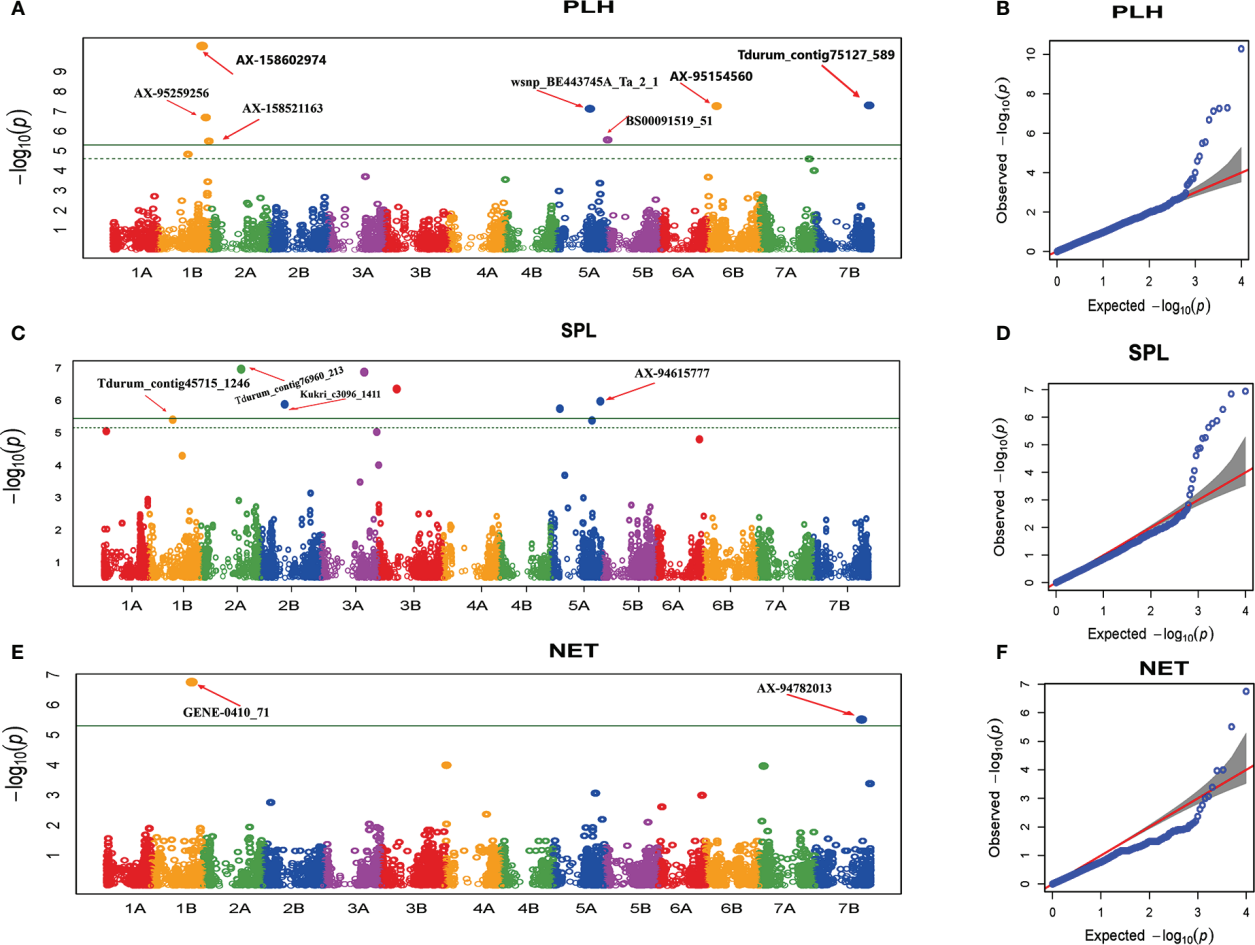
Manhattan and Q-Q plots of GWAS scan for plant architecture traits generated based on the combined data from five locations. PHT **(A, B)**, SPL **(C, D)**, NET **(E, F)**. For the Manhattan plots, the y-axis represents -log10 (p) of the traits, while the x-axis represents the relative positions of the SNP markers on each chromosome. PHT, Plant height; SPL, Spike length; NET, Number of effective tillers per plant.

The GWAS analysis revealed 14 MTAs significantly associated with plant architecture traits (**Table 4** and **Figure 6**). For PHT, six significant MTAs were detected (**Figure 6**). Among these, four (AX-158602974, and AX-95259256 on chromosome 1B, wsnp_BE443745A_Ta_2 on chromosome 5A, and BS00091519_51 on chromosome 5B) were previously reported (Zhang et al., 2012; Mengistu et al., 2016; Roncallo et al., 2017). The other two MTAs (AX-95154560 and Tdurum_contig75127_589 on chromosomes 1B and 7B, respectively) were novel. The six significant MTAs explained 1.2 to 23% of the variation in PHT recorded in this study.

The MTA analysis for spike length (SPL) revealed five significant MTAs. Among the significant MTAs, Tdurum_contig45715_1246 on chromosome 1B was previously identified (Giraldo et al., 2016). The remaining four MTAs (Kukri_c17062_618 and Tdurum_contig76960_213 on chromosome 2A, Kukri_c3096_1411 on chromosome 2B, and AX-94615777 on chromosome 5A) are novel (**Figure 6**). The proportion of the variation in SPL elucidated by the significant MTAs varied from 2.75% to 12.3%. For NET, GWAS revealed two (GENE-0410_71 and AX-94782013) significant MTAs on chromosome 1B and 7B, respectively (**Table 4** and **Figure 6**), which have not been previously reported. These MTAs explained 2.8% and 5.6% of the variation in NET, respectively.

#### 3.6.3 Marker trait association for grain yield and related traits

**Figure 7:**
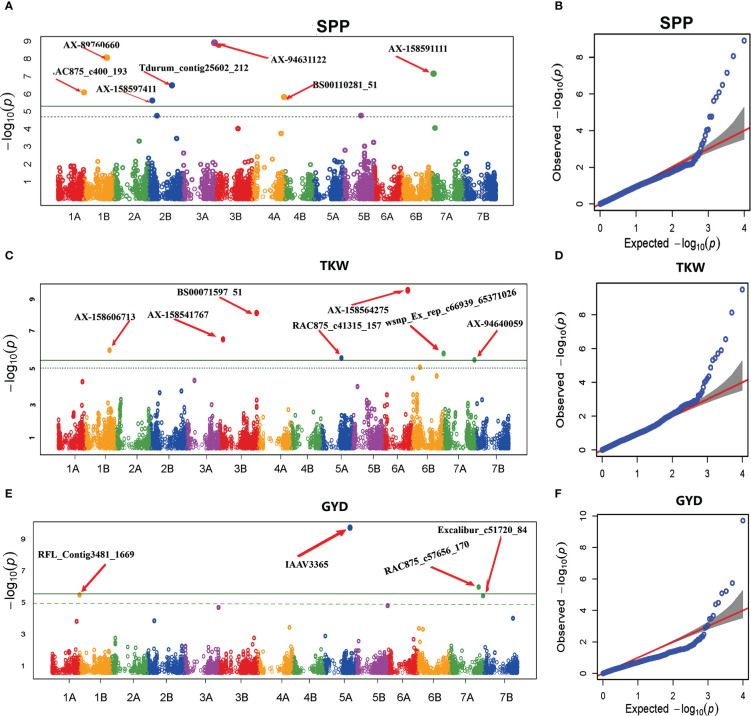
Manhattan and Q-Q plots of GWAS scan for SPP **(A, B)**, TKW **(C, D)**, and GYD **(E, F)** generated based on the combined data from five locations. For the Manhattan plots, the y-axis represents -log10 (p) of the traits, while the x-axis represents the relative positions of the SNP markers on each chromosome. SPP, Number of spikelets per spike; TKW, Thousand kernel weight; GYD, Grain yield.

GWAS for grain yield and yield-related traits evidenced 18 significant MTAs (**Table 6** and **Figure 7**). The association scan for SPP resulted in seven significant MTAs on chromosomes 1B, 2B, 3A, 4A, and 7A. The proportion of phenotypic variance explained by the associated MTAs ranged from 1.1% to 17%. Of the seven significant MTAs for SPP, four (AX-89760660, Tdurum_contig25602_212, BS00110281_51, and AX-158591111) were previously reported (Golabadi et al., 2011; Mengistu et al., 2016; Soriano et al., 2016; Kidane et al., 2017a; Roncallo et al., 2017; Abu-Zaitoun et al., 2018; Mangini et al., 2018), whereas the remaining three (RAC875_c400_193, AX-158597411, and AX-94631122) SNPs are novel.

**Figure 8:**
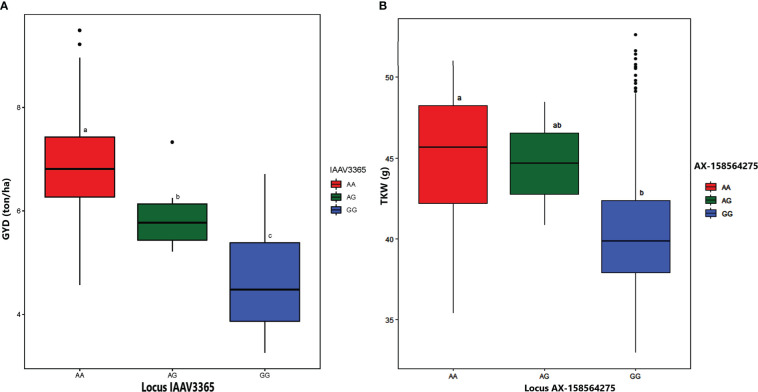
Boxplot depicting the effect of alleles located on locus IAAV3365 on grain yield **(A)** and locus AX-158564275 on thousand kernel weight **(B)**. The estimated mean BLUEs value was used to generate the boxplot to reveal the effects of respective alleles on grain yield and thousand kernel weight. Tukey´s HSD (Honestly Significant Difference) test was applied to see the differences of alleles in 420 durum wheat genotypes. The letters above the boxplot indicate the significant differences among the mean performance of genotypes carrying each allele at a probability level of *p< 0.05*.

For GYD, GWAS revealed four significant MTAs on chromosomes 1B, 5A, 5B, and 7A. The proportion of phenotypic variance explained by the significant MTAs ranged from 1.74% (RAC875_c57656_170 on chromosome 7A) to 44.95% (IAAV3365 on chromosome 5A). Alleles of the high signal MTAs (locus IAAV3365, A/G alleles) had a highly significant effect on grain yield (**Figure 8A**). The genotypes carrying allele A had higher average grain yield across the five environments as compared to genotypes carrying allele G. RAC875_c57656_170 was previously reported for GYD (Maccaferri et al., 2014), whereas the remaining three MTAs (IAAV3365, RFL_Contig3481_1669 on chromosome 1B, and Excalibur_c51720_84 on chromosome 5B) were newly detected in the present study.

The genome-wide association analysis identified seven significant MTAs for TKW on chromosomes 1B, 3B, 5A, 6A, and 7A. The phenotypic variance explained by the associated SNPs ranged from 1.05% to 10.6%. Among the MTAs significantly associated with TKW, two (AX-158606713 and wsnp_Ex_rep_c66939_65371026 on chromosomes 1B and 7A, respectively) were previously reported. However, the other five MTAs (BS00071597_51, AX-158541767, RAC875_c41315_157, AX-158564275, and AX-94640059 on chromosomes 3B, 5A, 6A, and 7A, respectively) were novel. The effect of alleles on locus AX-158564275 (A/G alleles) revealed a highly significant difference in TKW (**Figure 8B**). The genotypes with the allele A had high TKW compared to genotypes carrying allele G.

#### 3.6.4 Identification of putative novel MTAs and their underlying candidate genes

According to the LD decay information for each chromosome, a genomic region of ten Mbp around each significant SNP (five Mbp downstream and five Mbp upstream of the significant SNP) is considered to be a QTL. Significant SNPs within the ranges of 10 Mbp apart are considered to refer to the same QTL. Based on this approach, 37 QTLs were identified for the 44 significant MTAs (**Table 5**). The names of these QTLs (*q.gwas.01* to *q.gwas.*37) are provided in the first column of **Table 5**. Among the 37 QTLs, 16 were located in or near genomic regions previously reported for the corresponding traits (Golabadi et al., 2011; Maccaferri et al., 2014; Giraldo et al., 2016; Mengistu et al., 2016; Soriano et al., 2016; Kidane et al., 2017a; Abu-Zaitoun et al., 2018; Mangini et al., 2018; Roncallo et al., 2018), while 21 were novel (**Table 5**). Two QTLs for SPP (*q.gwas.15* and *q.gwas.20*) and one QTL (*q.gwas.08*) for TKW were previously described based on Ethiopian durum wheat germplasm (Mengistu et al., 2016; Kidane et al., 2017a). Genomic regions for five putative QTLs (*q.gwas.01, q.gwas.02, q.gwas.22, q.gwas.24*, and *q.gwas.30*) overlap with more than one trait evaluated in this study (**Table 5**). For example, four significant MTAs for DTH, DTM, SPP, and TKW were co-localized and hence were considered to be referring to the same QTL (*q.gwas.30*) (**Table 5**). The analysis of the sequences of these putative QTLs genomic regions based on durum wheat, the reference genome at the Ensemble Plants database, led to the identification of 774 potential candidate genes (**Supplementary Table 7**).

**Table 5:** Summary of putative quantitative trait loci (QTLs) identified for the nine phenotypic traits analyzed in the present study using Ethiopian durum wheat germplasm.

Putative QTL	Associated SNP	Chr	SNP position (bp)	TAPQTL
*q.gwas.01^a^*	IACX11338	1B	522669860	DTH
*q.gwas.02 ^a^*	RFL_Contig3481_1669	1B	4043159	GYD
*q.gwas.01 ^a^*	GENE-0410_71	1B	523053033	NET
*q.gwas.03 ^a^*	AX-158602974	1B	580658793	PHT
*q.gwas.04 ^a^*	AX-158521163	1B	669849432	PHT
*q.gwas.05*	AX-95259256	1B	629504430	PHT
*q.gwas.06 ^a^*	Tdurum_contig45715_1246	1B	314321199	SPL
*q.gwas.02*	RAC875_c400_193	1B	1547605	SPP
*q.gwas.07*	AX-89760660	1B	519060573	SPP
*q.gwas.08 ^a^*	AX-158606713	1B	546979073	TKW
*q.gwas.09 ^a^*	AX-94884567	2A	756029643	DTH
*q.gwas.10*	Kukri_rep_c73477_888	2A	70572673	DTM
*q.gwas.11*	Tdurum_contig76960_213	2A	492195805	SPL
*q.gwas.12*	Kukri_c17062_618	2A	522595273	SPL
*q.gwas.13*	Kukri_c3096_1411	2B	314134332	SPL
*q.gwas.14 ^a^*	AX-158597411	2B	99223728	SPP
*q.gwas.15*	Tdurum_contig25602_212	2B	546442999	SPP
*q.gwas.16*	AX-94631122	3A	723577013	SPP
*q.gwas.17 ^a^*	RAC875_c62223_86	3B	763192473	GFP
*q.gwas.18*	AX-158541767	3B	61267921	TKW
*q.gwas.19*	BS00071597_51	3B	803879943	TKW
*q.gwas.20 ^a^*	BS00110281_51	4A	724872914	SPP
*q.gwas.21*	IAAV3365	5A	548344620	GYD
*q.gwas.22*	wsnp_BE443745A_Ta_2_1	5A	439542987	PHT
*q.gwas.23*	AX-94615777	5A	529858969	SPL
*q.gwas.22*	RAC875_c41315_157	5A	431829169	TKW
*q.gwas.24 ^a^*	AX-109859693	5B	5172617	DTH
*q.gwas.24 ^a^*	BS00091519_51	5B	5174649	PHT
*q.gwas.25 ^a^*	AX-109869840	6A	603461435	DTM
*q.gwas.26*	AX-158564275	6A	528989018	TKW
*q.gwas.27*	wsnp_BE496986B_Ta_2_2	6B	568039035	DTH
*q.gwas.28*	Kukri_c60966_261	6B	693337622	GFP
*q.gwas.29*	AX-95154560	6B	120830636	PHT
*q.gwas.30 ^a^*	AX-158591262	7A	30075367	DTH
*q.gwas.30 ^a^*	Tdurum_contig49186_437	7A	32558067	DTM
*q.gwas.31*	Tdurum_contig12722_779	7A	44540113	DTM
*q.gwas.32*	RAC875_c57656_170	7A	614197852	GYD
*q.gwas.33 ^a^*	wsnp_Ex_c16045_24471413	5B	685974689	GYD
*q.gwas.30 ^a^*	AX-158591111	7A	33518205	SPP
*q.gwas.30 ^a^*	wsnp_Ex_rep_c66939_65371026	7A	6480158	TKW
*q.gwas.34*	AX-94640059	7A	686968079	TKW
*q.gwas.35*	Ku_c24482_1132	7B	155935903	DTH
*q.gwas.36*	AX-94782013	7B	604310198	NET
*q.gwas.37*	Tdurum_contig75127_589	7B	697951769	PHT

^a^Previously identified QTLs, Chr, chromosome; DTH, Days to heading; DTM, Days to physiological maturity; GFP, Grain filling; NET, Number of effective tillers per plant; GFP, Grain filling period; PHT, Plant height; SPL, Spike length; SPP, Number of spikelets per spike; TKW, Thousand kernel weight; GYD, Grain yield; TAPQTL, Traits associated with Putative QTL.

The significance of the candidate genes was evaluated by reviewing previously published genomic regions associated with the traits targeted in the present study (Zhang et al., 2012; Maccaferri et al., 2016; Maccaferri et al., 2019; Kidane et al., 2019; Mazzucotelli et al., 2020; Zhao et al., 2020). This resulted in 32 genes related to eight of the nine target traits in durum wheat (**Table 6**). The putative candidate genes *TRITD7Av1G01175*, *TRITD7Av1G017240*, and *TRITD7Av1G017550* (all located on chromosome 7A), which encode Growth-regulating factor, Zinc-finger CCCH domain protein TE, and NAC domain-containing protein, respectively, were associated with DTH and DTM. The genes *TRITD1Bv1G168480 and TRITD7Bv1G057630* encode WRKY transcription factor and Flowering Locus T/Terminal Flower 1-like protein, respectively, were reported to regulate DTH. The *TRITD1Bv1G000110* gene, on chromosome 1B, which encodes Tryptophan aminotransferase-related protein 2, was shown to be associated with SPP and GYD (**Table 6**).

**Table 6:** Summary of selected genes associated with some of the putative QTLs identified in the present study.

S/N	SNP	Chr	PQAG	Gene ID	GSS	GSE	Description of gene	Trait
1	IACX11338	1B	*q.gwas.01*	*TRITD1Bv1G168480*	523422292	523424581	WRKY transcription factor	
2	IACX11338	1B	*q.gwas.01*	*TRITD1Bv1G169970*	526873942	526876463	WRKY transcription factor	
3	IACX11338	1B	*q.gwas.01*	*TRITD1Bv1G170060*	527017659	527018012	WRKY DNA-binding protein 39 G	
4	AX-158591262	7A	*q.gwas.30*	*TRITD7Av1G011750*	20905388	20908162	Growth-regulating factor	
5	AX-158591262	7A	*q.gwas.30*	*TRITD7Av1G017240*	30780092	30782694	Zinc finger CCCH domain protein TE?	DTH
6	AX-158591262	7A	*q.gwas.30*	*TRITD7Av1G017550*	31546753	31548244	NAC domain-containing protein, putative	
7	Ku_c24482_1132	7B	*q.gwas.35*	*TRITD7Bv1G056500*	157854628	157855010	Seed maturation protein LEA 4	
8	Ku_c24482_1132	7B	*q.gwas.35*	*TRITD7Bv1G056720*	158562417	158562866	Zinc finger family protein	
9	Ku_c24482_1132	7B	*q.gwas.35*	*TRITD7Bv1G057630*	162519251	162520668	FLOWERING LOCUS T/TERMINAL FLOWER 1-like protein	
10	Ku_c24482_1132	7B	*q.gwas.35*	*TRITD7Bv1G058480*	164897716	164902189	Phosphate transporter PHO1-like protein	
11	AX-109869840	6A	*q.gwas.25*	*TRITD6Av1G220960*	603258174	603260717	Ethylene receptor	
12	Tdurum_contig49186_437	7A	*q.gwas.30*	*TRITD7Av1G011750*	20905388	20908162	Growth-regulating factor	DTM
13	Tdurum_contig49186_437	7A	*q.gwas.30*	*TRITD7Av1G017240*	30780092	30782694	Zinc finger CCCH domain protein TE?	
14	Tdurum_contig49186_437	7A	*q.gwas.30*	*TRITD7Av1G017550*	31546753	31548244	NAC domain-containing protein, putative	
15	Kukri_c60966_261	6B	*q.gwas.28*	*TRITD6Bv1G226900*	693249887	693252855	Receptor protein kinase, Putative	GFP
16	AX-158602974	1B	*q.gwas.03*	*TRITD1Bv1G189370*	580660944	580663298	Calcineurin B-like protein	
17	AX-158602974	1B	*q.gwas.03*	*TRITD1Bv1G189570*	580988293	580988886	Receptor-like protein kinase	PHT
18	AX-158602974	1B	*q.gwas.03*	*TRITD1Bv1G191400*	585136543	585142125	Zinc finger protein	
19	BS00091519_51	5B	*q.gwas.24*	*TRITD5Bv1G001780*	5178666	5198798	Cytochrome P450-like protein	
20	Kukri_c17062_618	2A	*q.gwas.12*	*TRITD2Av1G189490*	526757053	526762842	Acyl-CoA N-acyltransferase	
21	Kukri_c17062_618	2A	*q.gwas.12*	*TRITD2Av1G190600*	529454657	529455487	Ring finger protein, Putative	SPL
22	Kukri_c3096_1411	2B	*q.gwas.13*	*TRITD2Bv1G109560*	316041878	316042468	E3 ubiquitin-protein ligase	
23	RAC875_c400_193	1B	*q.gwas.02*	*TRITD1Bv1G000110*	327500	328114	Tryptophan aminotransferase-related protein 2	
24	AX-89760660	1B	*q.gwas.07*	*TRITD1Bv1G166820*	518699664	518701642	Zinc finger CCCH domain-containing protein 4	
25	AX-89760660	1B	*q.gwas.07*	*TRITD1Bv1G167110*	519057378	519065351	UDP-GLUCOSE PYROPHOSPHORYLASE 1	SPP
26	Tdurum_contig25602_212	2B	*q.gwas.15*	*TRITD2Bv1G184650*	545766407	545768972	ethylene-responsive transcription factor	
27	AX-94631122	3A	*q.gwas.16*	*TRITD3Av1G275580*	723577014	723578195	E3 ubiquitin-protein ligase	
28	AX-158606713	1B	*q.gwas.08*	*TRITD1Bv1G176830*	544878459	544879181	Ethylene-responsive factor-like transcription factor	
29	AX-158606713	1B	*q.gwas.08*	*TRITD1Bv1G177060*	545904571	545908993	E3 ubiquitin-protein ligase	TKW
30	AX-158606713	1B	*q.gwas.08*	*TRITD1Bv1G177540*	547614469	547614996	Blue copper protein	
31	RFL_Contig3481_1669	1B	*q.gwas.02*	*TRITD1Bv1G000110*	327500	328114	Tryptophan aminotransferase-related protein 2	GYD
32	IAAV3365	5A	*q.gwas.21*	*TRITD5Av1G205000*	550462923	550477847	ABC transporter	

Chr, chromosome; GSS, Gene Sequence starts; GSE, Gene sequence ends; APQ, Associated putative QTL; DTH, Days to heading; DTM, Days to physiological maturity; GFP, Grain filling period; PHT, Plant height; SPL, Spike length; SPP, Number of spikelets per spike; TKW, Thousand kernel weight; GYD, Grain yield.

## 4 Discussion

This study used GWAS to define durum wheat genomic regions associated with phenological, plant architecture, grain yield, and yield-related traits. Furthermore, analyses of population structure and linkage disequilibrium were carried out to increase the efficiency of detecting reliable marker-trait associations as well as identifying the genetic basis of those associations. The present study utilized a large number of diverse durum wheat landraces and cultivars, which were grown across diverse environments in Ethiopia. This facilitated the identification of novel SNP loci associated with nine durum wheat phenotypic traits, including grain yield and grain yield-related traits. The present study findings have significant implications for both the development of molecular markers for genomics-led breeding and for providing new insights into the architecture of genomic regions regulating various traits of interest in durum wheat. These could facilitate the improvement of grain yield and other desirable characteristics to support global food security.

To meet the growing demand for durum wheat grains as well as the challenges to its production brought about by the expanding environmental changes, it is imperative that the genetic resources of durum wheat, including landraces, modern cultivars, and breeding lines, be effectively utilized for breeding new cultivars (Kankwatsa et al., 2017; Maccaferri et al., 2019b; Kumar et al., 2020; Mazzucotelli et al., 2020). As such, identifying loci that regulate desirable traits in breeding programs helps to develop markers for marker-assisted breeding, thus contributing to food security (Garcia et al., 2019; Wang et al., 2019; Mérida-García et al., 2020).

The present study revealed highly significant contributions of genotypes, environments, and genotype by environment interactions to the phenotypic variations of the target traits (*p< 0.001*), which is consistent with the results of previous research on durum wheat (Mengistu et al., 2015; Mohammadi et al., 2018; Mekonnen et al., 2021). The observed high genotypic variance, genotypic coefficient of variation, and broad-sense heritability for TKW and GFP, strongly suggest that their variation is mainly due to heritable genetic differences among the landraces and cultivars. There was a low genotypic variance and genotypic coefficient of variation for GYD, indicating the challenges associated with improving this trait. Nevertheless, moderate to a high level of broad-sense heritability were recorded for all traits, meaning that a significant part of the observed variation is heritable and that the results agree with previous findings in durum wheat (Sukumaran et al., 2018; Alemu et al., 2020a).

The present study found that GYD had a moderate but significant (*p< 0.01*) positive correlation with SPP, and TKW, indicating that the simultaneous selection of desirable characteristics of these traits could lead to the improvement of grain yield in this crop. However, GYD negatively correlated with DTH and PHT, indicating that late-heading genotypes generally have lower grain yield than early-heading types. However, the early-heading types appear to have a more extended grain-filling period, as a very low but significant positive correlation was obtained for GYD versus DTM. TKW exhibited a moderate positive correlation with GYD, and GFP, implying that direct improvement of these traits may improve the former, which contributes to enhancing GYD. Conversely, TKW had a negative relation with DTH and DTM. Thus late-maturing cultivars will have a relatively low TKW.

The LD among the SNP marker pairs showed a sharp decline within the physical distance ranging from 2.02 to 10.4 Mbp, with an average of 4.26 Mbp. This decline in LD is far below the results of previous research using Ethiopian durum wheat landraces (Alemu et al., 2021b), which reported an average physical distance of 69.1 Mbp. Similarly, Mekonnen et al. (2021) found a higher mean LD decay (31.44 Mbp, r^2^ = 0.2) in their study on diverse Ethiopian bread wheat germplasm. This disparity could arise due to the type and density of markers, genomic regions the markers cover, and differences in the sample used in these studies. However, Fayaz et al., 2019 found a low LD decay (2–3 cM) of the A and B sub-genomes using Iranian durum wheat landraces at a critical r^2^ = 0.11. Likewise, Rufo et al. (2019) noted an LD decay ranging from 1 to 9 cM on A and B genomes from landraces and released cultivars of Mediterranean wheat. The fastest LD decay rate of an average physical distance of 2.02 Mbp was recorded for chromosome 7A.In contrast, the slowest was recorded for chromosome 4A (10.04 Mbp), which indicates the differences in recombination rates among different genomic regions of different chromosomes. On average, the A genome showed a more rapid LD decay than the B genome (**Supplementary Table 4**), and more substantial selection pressure could be partly caused in the A genome than in the B genome (Liu et al., 2019; Kumar et al., 2020). This result most likely confirms the impact of genetic drift, mutation, gene flow, recombination, the pressure of population selection, and historical events on both A and B genomes (Fayaz et al., 2019).

The population clustering inferred by STRUCTURE and PCA divided the genotypes into two sub-populations, similar to the results of earlier research (Wang et al., 2019; Alemu et al., 2020b; Kumar et al., 2020; Mekonnen et al., 2021). Based on Q-score values of STRUCTURE analysis (Q > 0.80), 96% of the landraces were pure, and 4% of the genotype were admixtures. The kinship matrix was used to estimate the family relatedness and to confirm the relation within the genotypes. Hence, the cumulative results from STRUCTURE, PCA, and kinship suggest adjusting the GWAS model to avoid bias arising from spurious associations, thereby reducing false-positive associations arising from co-ancestry. Moreover, FarmCPU, a robust statistical model for GWAS, adequately accounted for the spurious associations that arose from population structure, cryptic relatedness, and marker effects, as shown by Q-Q plots. Based on the five-test sites´ mean data, the GWAS revealed 44 MTAs. The SNPs associated with the target traits were distributed across the whole chromosome except chromosome 1A, which did not bear any significant MTAs.

Using GWAS, different genomic regions associated with grain yield were identified in the present study. The putative QTLs identified for this trait are *q.gwas.02* (0.3 – 5.8 Mbp) on chromosome 1B*, q.gwas.21* (544.7 – 554.9 Mbp) on chromosome 5A*, q.gwas.32* (609.2 – 619.2 Mbp) on chromosome 7A, and *q.gwas.33* (703.3 – 714.2 Mbp) on chromosome 7A. Among them, *q.gwas.02*, *q.gwas.21*, and *q.gwas.32* are novel QTLs, as these genomic regions have not previously been reported for their association with grain yield. The putative QTL *q.gwas.33* on chromosome 5B is co-localized within the same genomic region of a QTL reported by Maccaferri et al. (2014); Maccaferri et al. (2016) for grain yield and total root numbers, respectively using durum wheat recombinant inbred lines. The QTL regions of *q.gwas.33* is also identified for spikes per plant (Mengistu et al., 2016), kernel Fe content (Velu et al., 2017), kernels per spikelets (Peng et al., 2003), fusarium head blight resistance (Ghavami et al., 2011), yellow rust resistance (Liu et al., 2017a), and stem rust resistance (Letta et al., 2014). This QTL *q.gwas.33* is also associated with genes *TRITD5BvG245710* (myb-like protein X), *TRITD5Bv1G246830* (KH domain containing protein), *TRITD5Bv1G247760* (NBS-LRR disease resistance protein –like protein) and *TRITD5Bv1G246270* (Glycosyltransferase).

The QTL region of *q.gwas.02* (RFL_Contig3481_1669) for GYD, identified in this study, overlaps with QTLs for several traits such as total root number and length (Maccaferri et al., 2016), grain protein content and concentration (Suprayogi et al., 2009), spikes per plant (Mengistu et al., 2016), heading date (Maccaferri et al., 2008), grain filling period (Soriano et al., 2016), semolina yellowness (Colasuonno et al., 2017), grain yield per spike and grain yield (Roncallo et al., 2018), grain protein content (Giraldo et al., 2016) and fusarium head blight resistance (Ghavami et al., 2011). The overlapping of QTLs for several important traits in this genomic region indicates its significance in future durum wheat breeding for grain yield and end-use quality traits. The genomic region corresponding to QTL *q.gwas.21* on chromosome 5A (MTA for IAAV3365 SNP and GYD) is a novel major QTL for grain yield, explaining the largest proportion of phenotypic variance (r^2^ = 44.95%) as compared to all other putative QTLs reported here. This is a highly significant result of this study, which needs to be validated through further research, including fine mapping to pinpoint the gene(s) responsible for this QTL. Interestingly, the genomic region of this QTL overlaps with previously identified QTL for number of kernels per spike (Kidane et al., 2017a), yellow rust resistance (Liu et al., 2017b), threshing time (Tzarfati et al., 2014), leaf rust resistances (Aoun et al., 2016), and total root number (Maccaferri et al., 2016). Therefore, this genomic region is a key target region for the improving of durum wheat, for grain yield and threats of wheat arising due to the impacts of climate change. The *TRITD5Av1G205000* (an ABC transporter) gene is one of the potential candidate genes behind this QTL (*q.gwas.21*). This is because previous research indicated that ABC transporter genes affect grain formation in wheat during heading and also modulate the ripening of the heads (Wanke and Üner Kolukisaoglu, 2010; Walter et al., 2015).

The present study identified several novel QTLs for grain yield-related traits, SPP and TKW. Additional MTAs that confirmed previously identified genomic regions were also detected for these traits. The three novel putative QTLs for SPP are *q.gwas.07* (721.4–725.8 Mbp) on chromosome 1B, *q.gwas.15* (541.4–551.4 Mbp) on chromosome 2B and *q.gwas.16* (721.4–725.8 Mbp) on chromosome 3A. For TKW, five novel putative QTLs, i.e., *q.gwas.18* (58.8–68.0 Mbp) on chromosome 3B, *q.gwas.19* (803.2 – 812.9 Mbp) on chromosome 3B, *q.gwas.22* (431.8–442.1 Mbp) on chromosome 5A, *q.gwas.26* (524–534 Mbp) on chromosome 6A, and *q.gwas.34* (681.9–692 Mbp) on chromosome 7A were identified. Fine mapping of these genomic regions is required to identify the genes responsible for these QTLs for SPP and TKW. However, for four of the five novel QTLs for SPP, we were able to identify potential candidate genes, i.e., T*RITD1Bv1G000110* (Tryptophan aminotransferase-related protein 2), *TRITD1Bv1G167110* (UDP-Glucose Pyrophosphorylase 1), *TRITD2Bv1G184650* (ethylene-responsive transcription factor), and *TRITD3Av1G275580* (E3 ubiquitin-protein ligase). *TRITD2Bv1G184650* has been reported to regulate the initiation and development of spikelets in wheat, particularly when the temperature is low (Yu et al., 2021).

Several putative QTLs for SPP are identified here, i.e., *q.gwas.02* (0.3–5.8 Mbp), *q.gwas.14* (94–104.6 Mbp), *q.gwas.20* (721.4–725.8 Mbp), and *q.gwas.30* (2–35 Mbp) were found co-localized with previously reported QTLs for these traits on chromosomes 1B, 2B, 4A, and 7A, respectively (Golabadi et al., 2011; Mengistu et al., 2016; Kidane et al., 2017a; Roncallo et al., 2017; Mangini et al., 2018; Soriano et al., 2018; Li et al., 2019; Rahimi et al., 2019; Alipour et al., 2021). Similarly, putative QTLs for TKW were co-localized with QTLs previously identified, i.e.*, q.gwas.08* (MTA for AX-158606713) with a QTL identified based on Ethiopian durum wheat germplasm (Mengistu et al., 2016), and *q.gwas.30* with a QTL identified by Golabadi et al. (2011) based on F3 and F4 populations of durum wheat in Iran, and by Mangini et al. (2018) from a collection of tetraploid wheat grown in Southern Italy. The genomic region regarded as QTL *q.gwas.30* in this study was associated with four traits (DTH, DTM, SPP, TKW) (**Table 5**). This suggests that either the same gene with pleiotropic effects is involved in regulating these traits, or different genes in this genomic region regulate their corresponding traits or a combination of both. Thus, further research is required to identify common SNP markers representing the four traits in this genomic region and subsequent use in marker-assisted selection for improving the crop. Several genes encoding growth-regulating factor, seed maturation protein, phosphate transporter, phototropic-responsive NPH3 protein G, disease resistance protein RPM1, phosphate-responsive 1 family protein, E3-ubiquitin-protein ligase SINA-like 10, potassium transporter, and chloroplast envelope membrane protein, are among the likely candidates for the QTL q.gwas.30 (**Supplementary Table 7**).

The marker-trait association analysis conducted *via* GWAS discovered novel and previously identified genomic regions (putative QTLs) associated with DTH. Of these, *q.gwas.01* (522.7–528.6 Mbp of chromosome 1B)*, q.gwas.09* (753.4–757.6 Mbp of chromosome 2A), and *q.gwas.30* (2–35 Mbp Mbp of chromosome 3B) were previously reported for this trait (Golabadi et al., 2011; Roncallo et al., 2017; Mangini et al., 2018). These QTLs are significant at two or more test locations and hence can be considered stable MTAs across environments. The present findings also confirmed the results reported in previous studies for DTH on chromosomes 1B, 2A, and 3B (Kidane et al., 2017b; Ogbonnaya et al., 2017; Li et al., 2019; Rahimi et al., 2019; Wang et al., 2019). Hence, there is solid evidence of genes regulating DTH in these genomic regions. One of the novel putative QTLs for DTH is *q.gwas.35*, covering a 151.9–165.3 Mbp region on chromosome 7B. This genomic region contains the *TRITD7Bv1G057630* gene that encodes Flowering Locus T/Terminal Flower 1-like protein. Previous research on wheat, soybean, and Arabidopsis indicates that this gene is located in the region flanking *FT-D1*, a major gene regulating flowering in wheat, soybean, and Arabidopsis (Sun et al., 2019; Isham et al., 2021). Hence, if breeders aim to improve durum wheat for DTH, it is advisable to consider the QTL regions of *q.gwas.35* to get information related to DTH. Furthermore, as previously shown (Wu et al., 2008; Zhao et al., 2020), the *q.gwas.01* QTL region contains a potential candidate gene *TRITD1Bv1G168480* (WRKY). This gene involved in regulating leaf senescence. It is also known to have major roles at various stages of wheat development affecting productivity and product quality and was predicted to interact with DTH. Similarly, the *TRITD7Av1G017240* (zinc finger CCCH-type transcription factor) gene, which promotes wheat flowering, was also identified in this study. Hence, it would be worthwhile to conduct further research on this genomic region to identify the gene involved in *q.gwas.01* and to understand the relationship between leaf senescence and DTH in durum wheat.

A previously known genomic region and three novel genomic regions (putative QTLs) associated with DTM were found on chromosomes 2A, 6A, and 7A. These QTLs explained 2–33% of the variation in DTM. The QTL designated as *q.gwas.25* (601.5–615.3 Mbp of chromosome 6A) was reported in a previous study on wheat (Mangini et al., 2018). The novel putative QTLs are located on chromosomes 2A (67.5–70.6 Mbp; *q.gwas.10*) and 7A (2.1–35.1 Mbp; *q.gwas.30*, and 35.5–68.8 Mbp; *q.gwas.31*). The *TRITD6Av1G220960* (Ethylene receptor) gene, which is located within the genomic region of *q.gwas.25*, is a potential candidate gene for *q.gwas.25*. Previous research has suggested that ethylene receptors are most likely related to the duration of seed development and maturation; i.e., the duration embryo development (Hays et al., 2007). However, in previous findings in maize, grain yield increments were observed through ethylene signal reduction (Shi et al., 2015). Similar to the previous study (Han et al., 2021), *TRITD7Av1G011750* (growth-regulating factor), *TRITD6Av1G017240* (Zinc finger CCCH domain protein TE), and *TRITD7Av1G017550* (NAC domain-containing protein) genes have been found in the genomic regions of QTLs for DTH identified in this study, and are potential candidate genes for the corresponding QTLs. Studies have shown that these genes are mainly involved in regulating growth, development, biotic and abiotic stress adaptation in wheat, rice, and other crop plants and may also determine the variation in phenological traits in wheat and rice.

MTA analysis for PHT identified four putative QTLs, *q.gwas.03* (580.6–585.4 Mbp of chromosomes 1B), *q.gwas.04* (669.5–674.7 Mbp of chromosomes 1B) and *q.gwas24* (628.2–630.9 Mbp of chromosomes 5B), similar to the previous reports (Zhang et al., 2012; Roncallo et al., 2017). This study also found novel MTAs and putative QTLs on chromosome 1B (q.gwas.05; 628.2 – 630.9 Mbp), 5A (q.gwas.22; 431.8–442 Mbp), 6B (q.gwas.29; 119.6–121.5 Mbp), and 7B (q.gwas.37; 686.4–697.9 Mbp). These results show that all significant MTAs for PHT were on the B genome except one MTA on 5A. This may serve as an indicator of potential hotspot regions for genes associated with PHT. The *TRITD1Bv1G191400* (Zink finger protein) could be a candidate gene underlying the *q.gwas.03* QTL for PHT. Previous research revealed that this gene is significantly associated with improved salt tolerance and regulates stress resistance in wheat, Arabidopsis, and other plants (Ciftci-Yilmaz and Mittler, 2008; Ma et al., 2016). Other potential candidate genes for the PHT QTLs include *TRITD1Bv1G189370* (encoding Calcineurin B-like protein), *TRITD1Bv1G189570* (encoding Receptor-like protein kinase), and *TRITD5Bv1G0001780* (Cytochrome P450-like protein).

The present study confirmed previously reported QTL for SPL on chromosome 1B (*q.gwas.06*; 314.3–318.8 Mbp), which was previously reported by Giraldo et al. (2016). Likewise, putative QTL *q.gwas.11* on chromosome 2A (489.5–492.5 Mbp), QTL *q.gwas.12* on chromosome 2A (522.4–534.5 Mbp), QTL *q.gwas.13* on chromosome 2B (185.5–195.6 Mbp), and QTL *q.gwas.23* on chromosome 5A (526.3–534.1Mbp) were found for this trait. These are likely to be novel QTLs since the corresponding genomic regions are not associated with SPL in previous studies. The potential candidate genes for the SPL QTLs include *TRITD2Av1G189490* (*encoding* Acyl-CoA N-acyltransferase), *TRITD2Av1G190600* (encoding Ring finger protein), and *TRITD2Bv1G109560* (encoding E3 ubiquitin-protein ligase). A report from a previous study revealed that E3 ubiquitin proteins have a potential role in modulating crop productivity by influencing growth, development, and important agronomic traits (Varshney and Majee, 2022).

## 5 Conclusions

In the present study, we evaluated the diverse germplasm of Ethiopian durum wheat using multi-environment trials (MET) data that are genotyped with the Illumina Infinium 25k wheat SNP array to unravel genomic regions associated with its phenological and plant architecture traits as well as grain yield and yield related traits using GWAS. The GWAS identified 44 significant MTAs, including 26 novel genomic regions. The combined analysis of variance revealed significant effects of genotype, environment, and genotype-by-environment interaction on the target traits. The study also confirmed several previously reported QTLs. The identification of a large number of novel QTLs in this study indicates the presence of novel alleles of the genes underlying these QTLs, which probably confirms the distinctness of the Ethiopian durum wheat gene pool from other durum wheat gene pools. The major significant QTLs, such as *q.gwas.21* (for SNP IAAV3365, stable across location) that explained 44.95% of the variation in grain yield, *q.gwas.10* (for SNP Kukri_rep_c73477_888) that explained 32.9% of the variation in days to maturity and *q.gwas.28* (for SNP Kukri_c60966_261) that explained 30.7% of the variation in the grain-filling period are the key findings of this study. Additional research is needed to validate these key findings, including fine mapping to determine the underlying genes and their subsequent functional analysis. The addition of SNP markers associated with the target traits of this study is highly beneficial for genomic-led breeding of durum wheat.

The results could empower the sustainability of durum breeding by unlocking genomic regions governing complex plant characteristics. Most importantly, the results obtained in the present study could contribute a major role in understanding the durum wheat genome and improving genetic resources for breeding this crop, which in turn, supports global food security. The newly identified genes will also advance the understanding of genomic regions associated with essential characteristics used in durum wheat breeding. The identified novel variants suggest a potential use of Ethiopian durum wheat in durum wheat marker-assisted breeding. The study also provided new insight into the genetic architecture of grain yield and related traits. It indicated the potential of the diverse Ethiopian durum wheat gene pool for future improvement programs. Hence, the identified MTAs and candidate genes could be used to understand the genetic basis of genomic regions of important traits and to accelerate the development of new cultivars with high grain yield and agronomically essential traits *via* precision breeding. In addition, the identified MTAs could be used in marker-assisted breeding, fine mapping, and cloning of the underlying genomic regions and putative QTLs in durum wheat germplasm.

## Data availability statement

The genotypic data presented in this study were generated using a commercially available Illumina Infinium 25k wheat SNP array whose details can be found at https://www.traitgenetics.com/index.php/service-products. The genotypic data of the 420 genotypes studied can be obtained upon request.

## Author contributions

Conceptualization: BM, KT, RO, MG, EJ, TH, Methodology: BM, MG, KT, RO, EJ, TH, CH, Data curation: BM, Formal analysis: BM, Visualization: BM, MG, Investigation: BM, MG, RO, EJ, Resources: KT, RO, EJ, MG, TH, Funding acquisition: KT, RO, EJ, MG, TH, Project administration: KT, RO, MG, EJ, TH, Supervision: KT, RO, EJ, MG, TH, CH, FH, Writing original draft: BM, Writing-review, and editing: BM, RO, EJ, MG, KT, TH, CH, and FH. All authors have read and approved the final version of the manuscript.

## Supplementary material

Supplementary Table 2 Description of mean monthly weather information of test sites during crop growing season.

Supplementary Table 5 Computed physical distance at which mean linkage disequilibrium decay drops below at cut off (r^2^ = 0.2) value on pairwise comparison of SNP markers.

Supplementary Table 6 GWAS complete output for phenological, plant architecture, grain yield, and yield-related traits across five test sites and combined data. “Trait” indicate measured phenotypic data including; DTH, Days to heading; DTM, Days to physiological maturity; NET, Number of effective tillers per plant; GFP, Grain filling period; PHT, Plant height; SPL, Spike length; SPP, Number of spikelets per spike; TKW, Thousand kernel weight; and GYD, Grain yield. Loc, test sites, AK, CD, HO, KU, and SN are Akaki, Chefe Donsa, Holeta, Kulumsa, and Sinana test sites, respectively. CO; reports combined environments data; CHR, chromosome; SNP, Single nucleotide polymorphisms; POS, Physical position; MAF, minor allele frequency; P.value, reports the significance of the nominal tests and PVE, reports phenotypic variance explained. This **Supplementary Table** is also presented in Excel format.

Supplementary Table 7 High-confidence candidate genes were identified through marker-trait association analysis to associate with phenological, plant architecture, grain yield, and yield-related traits collected at five test sites. “Trait” indicates measured phenotypic data including; DTH, Days to heading; DTM, Days to physiological maturity; NET, Number of effective tillers per plant; GFP, Grain filling period; PHT, Plant height; SPL, Spike length; SPP, Number of spikelets per spike; TKW, Thousand kernel weight; and GYD, Grain yield. MTAs, Marker trait associations; SNPs, Single nucleotide polymorphisms. This **Supplementary Table** is also presented in Excel format.

Supplementary Figure 1 Genome-wide LD decay plot over total physical distance based on 10,045 SNP markers. The yellow curve represents the model that fits LD decay. The solid red line represents the arbitrary threshold for no LD used (*r^2^* = 0.3). The light green line indicates the intersection between the critical and the map distance to determine QTL confidence intervals.

Supplementary Figure 2 The circular Manhattan and Q-Q plots of GWAS results for DTH on panels **(A, B)**, and DTM on panels **(C, D)** were produced using each test site and combined data across test sites. The circular Manhattan plots represent the relative positions of the SNP markers on each chromosome in a circular manner. To view the significant MTAs results, move from outside to the center of each circle, rotating (rounding) through each circle starting from FarmCPU.CO.DTH, followed by FarmCPU.SN.DTH, FarmCPU.KU.DTH, FarmCPU.HO.DTH, FarmCPU.CD.DTH, and FarmCPU.AK.DTH to the center of the circle in sequential order and follow a similar approach for DTM. The name of test sites as stated here is presented in the center of circle. For the QQ–plots, Y-axis represents observed–log10 (p-value), and the X-axis represents expected–log10 (p-value) under the assumption that the *p*-values follow a normal distribution. The dotted lines indicate the 95% confidence interval assuming the null hypothesis of no association between the SNP and trait. DTH refers to days to heading, and DTM refers to days to physiological maturity. CO; Combined data across five environments, SN: Sinana site, KU: Kulumsa site, HO; Holeta site, CD: Chefe Donsa site, and AK: Akaki sites. The Circular Manhattan and Q-Q plots of GWAS results for GYD on panel **(E, F)**, TKW on panel **(G, H)**, and SPP on panel **(I, J)** were plotted using data from each test site and combined data from all test sites, respectively. For the circular manhattan plot, follow a similar approach for these traits as in **Figure S2A** for all test sites and traits. The assumption of QQ–plots in also applies here. GYD, Grain yield, TKW, Thousand-kernel weight, and SPP, number of spikelets per spike. CO; Combined data across five environments, SN: Sinana site, KU: Kulumsa site, HO; Holeta site, CD: Chefe Donsa site, and AK: Akaki sites. The Circular Manhattan and Q-Q plots of GWAS results for PHT on panel **(K, L)**, NET on panel **(M, N)**, and SPL on panel **(O, P)** were plotted using data from each test site and combined data from all test sites, respectively. For the circular manhattan plot, follow a similar approach for these traits as in **Figure S 2A** for all test sites and traits. For the QQ–plots, apply the assumption in a. SPL, Spike length; NET, Number of effective tillers per plant; and PHT, Plant height, and SPP. CO; Combined data across five environments, SN, Sinana site; KU, Kulumsa site; HO, Holeta site; CD, Chefe Donsa site; and AK, Akaki sites.
