# Supplementary material for: Marker-trait association analyses revealed major novel QTLs for grain yield and related traits in durum wheat
Source: Front Plant Sci. 2023 Jan 26;13:1009244. doi: 10.3389/fpls.2022.1009244 (PMC9909559; doi:10.3389/fpls.2022.1009244)
Supplement: Supplementary Table 2 — Description of mean monthly weather information of test sites during crop growing season. [file Table_2.docx]

**Supplementary Table 2.** Description of mean monthly weather information of test sites during crop growing season

|  | **Holeta** | | | | **Sinana** | | | **Akaki** | | | | **Chefe donsa** | | |
| --- | --- | --- | --- | --- | --- | --- | --- | --- | --- | --- | --- | --- | --- | --- |
|  | **MinT** | **MaxT** | **RF** | **RH** | **MinT.** | **MaxT.** | **RF** | **MaxT.** | **MinT** | **RF** | **RH** | **MaxT.** | **MinT.** | **RF** |
| **January** | 10.6 | 24.0 | 22.8 | 58.7 | 9.1 | 23.9 | 0.0 | 26.1 | 11.9 | 27.8 | 44.3 | 23.4 | 8.2 | 0.0 |
| **February** | 7.9 | 25.7 | 2.6 | 46.0 | 9.2 | 22.5 | 16.6 | 27.3 | 10.2 | 0.0 | 31.8 | 25.4 | 11.4 | 0.0 |
| **March** | 7.2 | 26.6 | 16.2 | 68.4 | 8.8 | 22.2 | 53.8 | 29.0 | 13.9 | 26.7 | 49.9 | 27.1 | 12.7 | 0.0 |
| **April** | 10.6 | 24.9 | 80.1 | 60.1 | 8.7 | 23.5 | 112.9 | 27.2 | 13.9 | 74.8 | 71.3 | 27.1 | 13.2 | 20.9 |
| **May** | 9.2 | 23.5 | 109.2 | 62.7 | 8.8 | 22.6 | 80.1 | 29.7 | 13.8 | 13.6 | 60.5 | 26.6 | 13.3 | 34.6 |
| **June** | 9.5 | 22.5 | 166.4 | 68.6 | 8.7 | 22.8 | 24.6 | 27.7 | 14.1 | 121.7 | 79.4 | 24.2 | 12.0 | 80.4 |
| **July** | 10.4 | 20.8 | 249.0 | 73.2 | 8.2 | 21.2 | 2.5 | 24.8 | 13.8 | 235.8 | 82.9 | 22.7 | 11.7 | 358.2 |
| **August** | 10.0 | 19.6 | 356.1 | 76.1 | 8.7 | 20.8 | 105.5 | 25.2 | 13.8 | 171.8 | 82.1 | 21.5 | 11.8 | 233.0 |
| **September** | 6.5 | 20.5 | 97.0 | 69.4 | 9.0 | 21.3 | 147.1 | 26.9 | 13.3 | 71.4 | 65.7 | 22.3 | 11.8 | 202.9 |
| **October** | 3.8 | 23.9 | 7.8 | 74.3 | 8.2 | 23.2 | 160.4 | 25.9 | 13.3 | 99.9 | 72.1 | 21.0 | 10.8 | 23.9 |
| **November** | 6.0 | 22.1 | 28.2 | 62.5 | 7.5 | 21.5 | 106.4 | 25.3 | 12.6 | 10.9 | 75.3 | 21.4 | 10.6 | 39.4 |
| **December** | 3.8 | 23.1 | 3.6 | 70.3 | 6.4 | 22.5 | 0.0 | 25.9 | 9.9 | 0.0 | 50.9 | 20.3 | 9.4 | 13.1 |
| **Annual Mean** | 8.0 | 23.1 | 94.9 | 65.9 | 8.4 | 22.3 | 67.5 | 26.8 | 12.9 | 71.2 | 63.9 | 23.6 | 11.4 | 83.9 |

NB. MinT = Minimum monthly temperature, MaxT. = Mean monthly temperature, RF = mean monthly rainfall, RH = relative humidity
