# Supplementary material for: Marker-trait association analyses revealed major novel QTLs for grain yield and related traits in durum wheat
Source: Front Plant Sci. 2023 Jan 26;13:1009244. doi: 10.3389/fpls.2022.1009244 (PMC9909559; doi:10.3389/fpls.2022.1009244)
Supplement: Supplementary file 3 [file Table_3.docx]

**Supplementary Table 3.** The Mean square (MS) of each source of variation for the nine phenotypic traits measured for 420 Ethiopian durum wheat genotypes grown in five environments.

| Traits | Source of variation | | | | | | | |  |
| --- | --- | --- | --- | --- | --- | --- | --- | --- | --- |
|  | G ^z^ (D_f_ = 419) | E (D_f_ = 4) | R ( D_f_ = 1) | B (D_f_ = 38) | G × E  (D_f_ =1676) | R x E  (D_f_ = 4) | B x R x E  (D_f_ = 152) | Residual (D_f_ = 1905) | |
| DTH | 108.3*** | 15405.7*** | 460.0** | 6.0** | 9.5*** | 232.5*** | 12.6** | 6.0 | |
| DTM | 21.9*** | 6233.7** | 632.9** | 4.5** | 7.3*** | 268.1** | 14.2** | 3.9 | |
| GFP | 91.8*** | 18015.0** | 12.2ns | 17.4** | 13.4** | 546.9** | 22.9* | 9.0 | |
| PHT | 425.0*** | 250806.0** | 6555* | 47.7ns | 87.0** | 758.0** | 167.0** | 58.0 | |
| SPL | 11.0*** | 1136.7** | 150.6** | 2.24** | 1.4** | 24.3** | 2.2*** | 0.9 | |
| SPP | 10.3*** | 4951.3** | 12.3** | 6.8** | 2.7** | 42.9** | 4.5*** | 1.8 | |
| NET | 4.7** | 2.23** | 878.63** | 8.18** | 2.2** | 38.0** | 7.5** | 1.9 | |
| TKW | 167.6** | 8401.7** | 230.8** | 12.66** | 15.9** | 175.4** | 14.9** | 7.0 | |
| GYD | 10.7*** | 3165.5** | 8.5* | 0.82** | 3.0*** | 22.3** | 1.5** | 0.6 | |

^z^ G = Genotypes; E = Environments; R = Replications; B = Incomplete block nested in replications; Df = Degree of freedom; DTH = Days to heading; DTM = Days to physiological maturity; GFP = Grain filling period; PHT = Plant height; SPL = Spike length; SPP = Number of spikelets per spike; NET = Number of effective tillers; TKW = Thousand kernel weight; GYD = Grain yield. ***, ***,* * indicate significance at *P* < 0.001, *P* < 0.01, and *P* < 0.05. ns = *P* > 0.05
