# Supplementary material for: Marker-trait association analyses revealed major novel QTLs for grain yield and related traits in durum wheat
Source: Front Plant Sci. 2023 Jan 26;13:1009244. doi: 10.3389/fpls.2022.1009244 (PMC9909559; doi:10.3389/fpls.2022.1009244)
Supplement: Supplementary file 4 [file Table_4.docx]

**Supplementary** **Table 4**. The combined mean, estimate of variance components, and broad-sense heritability (H^2^) for nine phenotypic traits of the diverse genotypes of durum wheat grown in five environments.

| Traits | Estimated variance component ^Z^ | | | | | | | |
| --- | --- | --- | --- | --- | --- | --- | --- | --- |
|  | Pooled Mean | σ^2^_g_ | σ^2^_g×xl_ | σ^2^_e_ | σ^2^_r_ | GCV | PCV | H^2^ |
| DTH | 72.60 | 9.33 | 0.78 | 19.34 | 6.40 | 35.85 | 37.78 | 0.90 |
| DTM | 136.00 | 7.00 | 1.03 | 7.09 | 3.92 | 22.69 | 24.11 | 0.89 |
| GFP | 63.40 | 56.75 | 1.42 | 20.91 | 8.93 | 94.61 | 95.94 | 0.97 |
| PHT | 96.30 | 33.02 | 7.29 | 343.67 | 5.89 | 58.56 | 62.20 | 0.89 |
| SPL | 7.94 | 7.47 | 1.48 | 1.32 | 8.78 | 97.00 | 106.99 | 0.82 |
| SPP | 17.50 | 7.76 | 2.40 | 5.85 | 1.76 | 66.55 | 72.21 | 0.85 |
| NET | 6.00 | 4.15 | 0.00 | 0.58 | 1.83 | 83.17 | 84.98 | 0.96 |
| TKW | 40.90 | 74.70 | 3.80 | 9.80 | 7.00 | 135.10 | 137.50 | 0.97 |
| GYD | 6.70 | 1.00 | 1.13 | 3.74 | 0.60 | 39.38 | 49.78 | 0.63 |

^z^ σ^2^_g_ = Genotypic variance; σ^2^_g×e_ = Genotype by environment interaction variance; σ^2^e = Environment variance; σ^2^_r_ = Error variance; GCV = Genotypic coefficient of variation; PCV = Phenotypic coefficient of variations; DTH = Days to heading; DTM = Days to physiological maturity; GFP = Grain filling period; PHT = Plant height (cm); SPL = Spike length (cm); GYD = Grain yield (t ha^-1^); TKW = Thousand kernel weight (g); SPP = Number of spikelet per spike
