# Supplementary material for: Marker-trait association analyses revealed major novel QTLs for grain yield and related traits in durum wheat
Source: Front Plant Sci. 2023 Jan 26;13:1009244. doi: 10.3389/fpls.2022.1009244 (PMC9909559; doi:10.3389/fpls.2022.1009244)
Supplement: Supplementary Table 5 — Computed physical distance at which mean linkage disequilibrium decay drops below at cut off (r2 = 0.2) value on pairwise comparison of SNP markers. [file Table_5.docx]

**Supplementary Table 5**. Computed physical distance at which mean linkage disequilibrium decay drops below at cut off (r^2^ = 0.2) value on pairwise comparison of SNP markers

| CHR | Dist_bp | Dist_Mbp | r^2^ value at cut off 0.3 |
| --- | --- | --- | --- |
| 1A | 3247768 | 3.25 | 0.300046 |
| 1B | 3511993 | 3.51 | 0.300641 |
| 2A | 3753990 | 3.75 | 0.300379 |
| 2B | 3473549 | 3.47 | 0.300048 |
| 3A | 5611433 | 5.61 | 0.300006 |
| 3B | 4424889 | 4.42 | 0.30038 |
| 4A | 10035133 | 10.04 | 0.300016 |
| 4B | 2730382 | 2.73 | 0.300068 |
| 5A | 6160825 | 6.16 | 0.300014 |
| 5B | 5800674 | 5.80 | 0.300054 |
| 6A | 4958129 | 4.96 | 0.300203 |
| 6B | 5277017 | 5.27 | 0.300760 |
| 7A | 2017591 | 2.02 | 0.300090 |
| 7B | 3723824 | 3.72 | 0.300013 |
| A genome | 3247768 | 3.25 | 0.300006 |
| B genome | 3511993 | 3.51 | 0.300013 |
| Genome mean | 4267724 | 4.27 |  |
