# Supplementary figures and images for: Marker-trait association analyses revealed major novel QTLs for grain yield and related traits in durum wheat
Source: Front Plant Sci. 2023 Jan 26;13:1009244. doi: 10.3389/fpls.2022.1009244 (PMC9909559; doi:10.3389/fpls.2022.1009244)

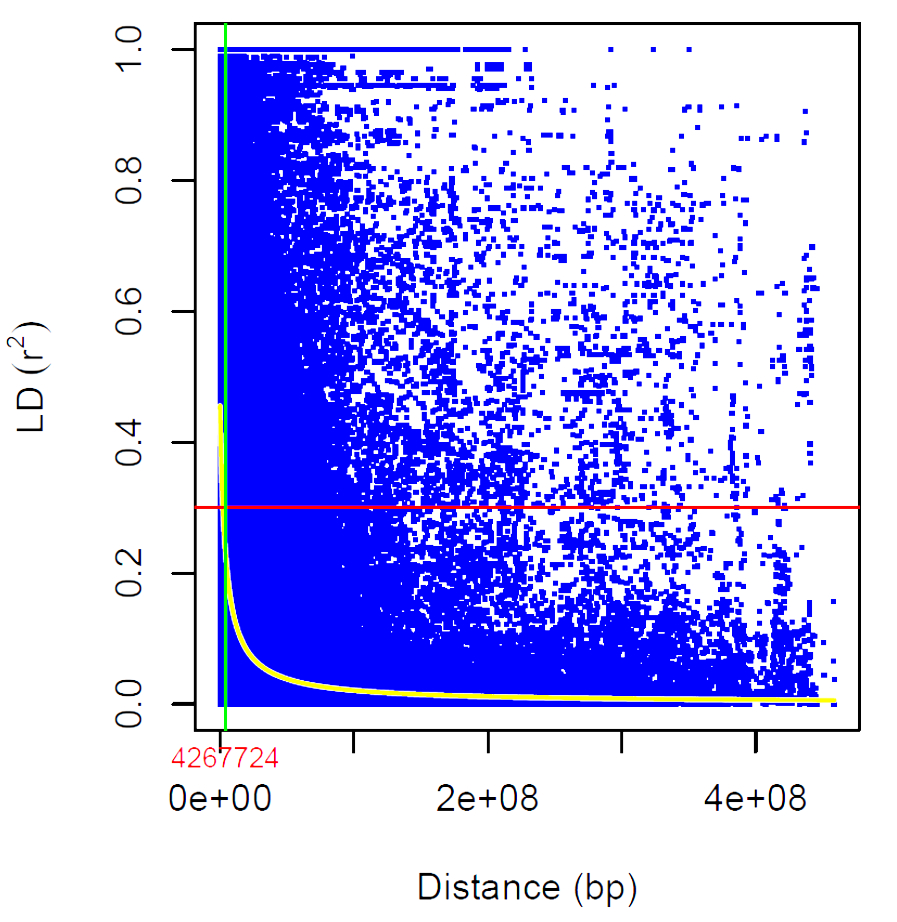

Supplement: Supplementary Figure 1 — Genome-wide LD decay plot over total physical distance based on 10,045 SNP markers. The yellow curve represents the model that fits LD decay. The solid red line represents the arbitrary threshold for no LD used (r2 = 0.3). The light green line indicates the intersection between the critical and the map distance to determine QTL confidence intervals. [file Image_1.jpeg]

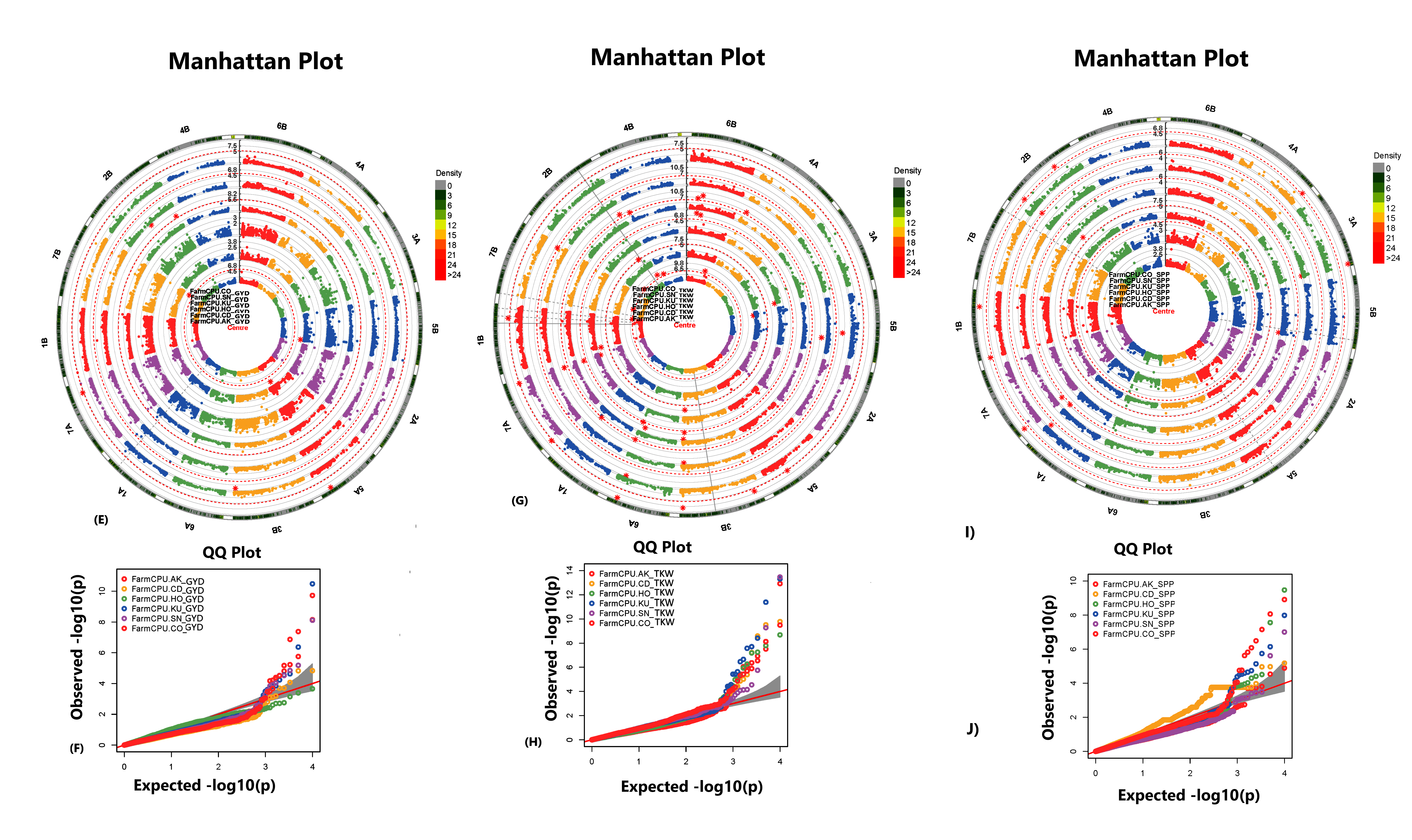

Supplement: Supplementary Figure 2 — The circular Manhattan and Q-Q plots of GWAS results for DTH on panels (A, B), and DTM on panels (C, D) were produced using each test site and combined data across test sites. The circular Manhattan plots represent the relative positions of the SNP markers on each chromosome in a circular manner. To view the significant MTAs results, move from outside to the center of each circle, rotating (rounding) through each circle starting from FarmCPU.CO.DTH, followed by FarmCPU.SN.DTH, FarmCPU.KU.DTH, FarmCPU.HO.DTH, FarmCPU.CD.DTH, and FarmCPU.AK.DTH to the center of the circle in sequential order and follow a similar approach for DTM. The name of test sites as stated here is presented in the center of circle. For the QQ–plots, Y-axis represents observed–log10 (p-value), and the X-axis represents expected–log10 (p-value) under the assumption that the p-values follow a normal distribution. The dotted lines indicate the 95% confidence interval assuming the null hypothesis of no association between the SNP and trait. DTH refers to days to heading, and DTM refers to days to physiological maturity. CO; Combined data across five environments, SN: Sinana site, KU: Kulumsa site, HO; Holeta site, CD: Chefe Donsa site, and AK: Akaki sites. The Circular Manhattan and Q-Q plots of GWAS results for GYD on panel (E, F), TKW on panel (G, H), and SPP on panel (I, J) were plotted using data from each test site and combined data from all test sites, respectively. For the circular manhattan plot, follow a similar approach for these traits as in Figure S2A for all test sites and traits. The assumption of QQ–plots in also applies here. GYD, Grain yield, TKW, Thousand-kernel weight, and SPP, number of spikelets per spike. CO; Combined data across five environments, SN: Sinana site, KU: Kulumsa site, HO; Holeta site, CD: Chefe Donsa site, and AK: Akaki sites. The Circular Manhattan and Q-Q plots of GWAS results for PHT on panel (K, L), NET on panel (M, N), and SPL on panel (O, P) were plotted usi [file Image_2.tiff]

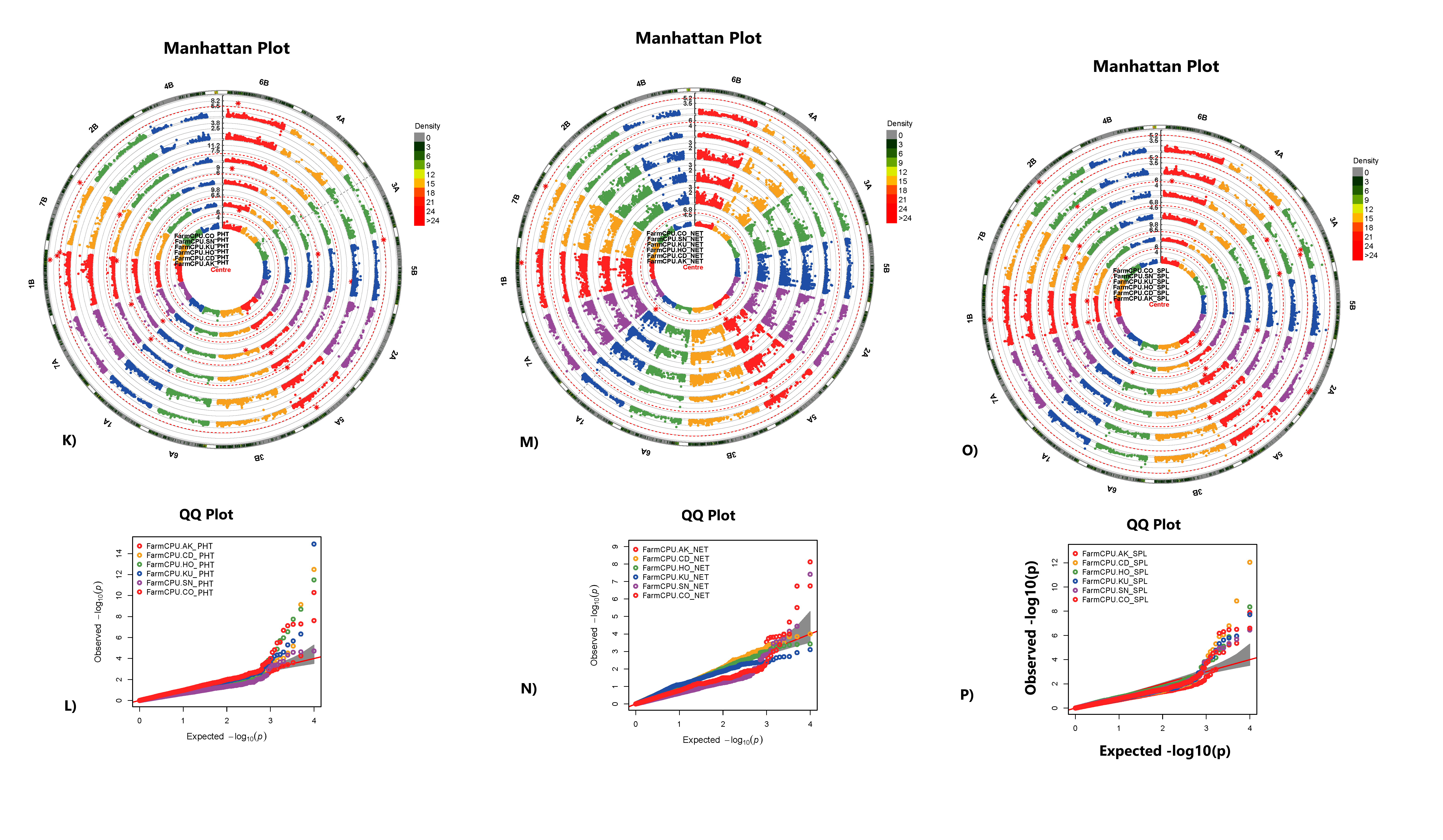

Supplement: Supplementary file 10 [file Image_3.tiff]

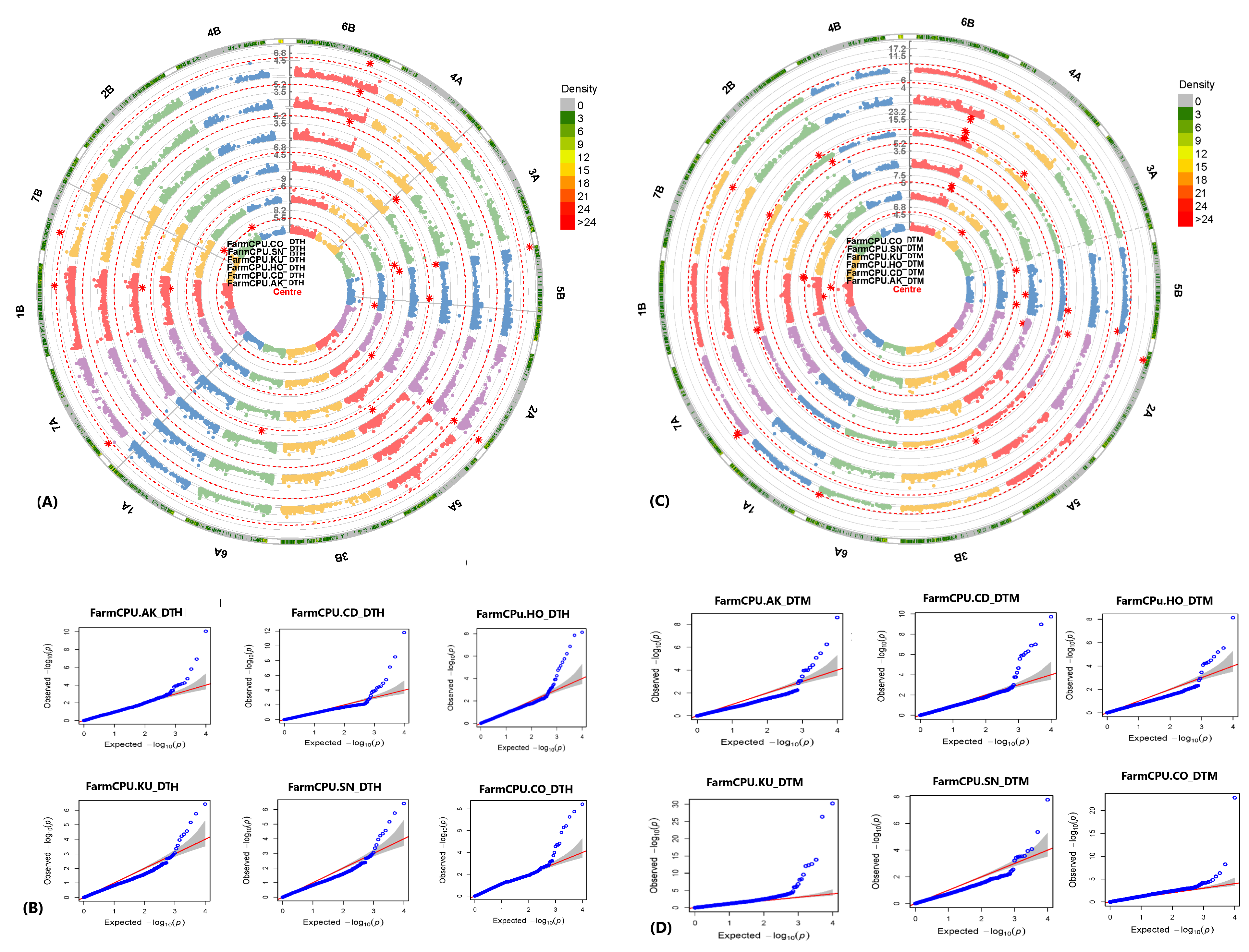

Supplement: Supplementary file 11 [file Image_4.tiff]
